# Mouse HSA^+^ immature cardiomyocytes persist in the adult heart and expand after ischemic injury

**DOI:** 10.1371/journal.pbio.3000335

**Published:** 2019-06-27

**Authors:** Mariana Valente, Tatiana Pinho Resende, Diana Santos Nascimento, Odile Burlen-Defranoux, Francisca Soares-da-Silva, Benoit Dupont, Ana Cumano, Perpétua Pinto-do-Ó

**Affiliations:** 1 Instituto de Investigação e Inovação em Saúde (i3S), Universidade do Porto, Porto, Portugal; 2 Instituto Nacional de Engenharia Biomédica (INEB), Universidade do Porto, Porto, Portugal; 3 Instituto de Ciências Biomédicas Abel Salazar (ICBAS), Universidade do Porto, Porto, Portugal; 4 Unit for Lymphopoiesis, Immunology Department, INSERM U1223, Institut Pasteur, Paris, France; 5 Université Paris Diderot, Sorbonne Paris Cité, Cellule Pasteur, Paris, France; 6 Beckman Coulter, France S.A.S, Villepinte, France; University of Cambridge, UNITED KINGDOM

## Abstract

The assessment of the regenerative capacity of the heart has been compromised by the lack of surface signatures to characterize cardiomyocytes (CMs). Here, combined multiparametric surface marker analysis with single-cell transcriptional profiling and in vivo transplantation identify the main mouse fetal cardiac populations and their progenitors (PRGs). We found that CMs at different stages of differentiation coexist during development. We identified a population of immature heat stable antigen (HSA)/ cluster of differentiation 24 (CD24)^+^ CMs that persists throughout life and that, unlike other CM subsets, actively proliferates up to 1 week of age and engrafts cardiac tissue upon transplantation. In the adult heart, a discrete population of HSA/CD24^+^ CMs appears as mononucleated cells that increase in frequency after infarction. Our work identified cell surface signatures that allow the prospective isolation of CMs at all developmental stages and the detection of a subset of immature CMs throughout life that, although at reduced frequencies, are poised for activation in response to ischemic stimuli. This work opens new perspectives in the understanding and treatment of heart pathologies.

## Abstract 2

This study identifies HSA/CD24 as a unique marker for immature cardiomyocytes, showing that HSA-positive cardiomyocytes persist throughout life, proliferating within the first week after birth and responding to myocardial infarction in the adult by expanding through cell division.

## Introduction

The cell types that form the mammalian heart have diverse developmental origins and temporal differentiation [1]. In the mouse, cardiomyocytes (CMs) are initially specified by embryonic day (E) 7.5 from the first set of cardiogenic progenitors (PRGs; first heart field) [2] followed by the incoming cells from the second heart field (SHF) [3]. At E 9.5 (looping-heart stage), the heart is divided into primitive atria (PAt), primitive ventricle (PVt), and outflow tract (OFT; future great vessels and atrioventricular junction [GV-AVJ]) and is composed of CMs and endocardial cells (EndoCs) that form the endocardium. Cells migrating from the peripheral tissues shape the final heart morphology. From E 9.5 to E 11.0, epicardial cells (EpiCs) derive from the proepicardial organ and coat the heart surface (epicardium). A fraction of EpiCs undergo epithelial-to-mesenchymal transition (EMT), giving rise to epicardial-derived cells (EPDCs), which migrate into the myocardium and generate peri-vascular smooth muscle cells (SMCs) [4] and fibroblasts (FBs) [5,6].

CMs contribute to heart growth [7,8] through extensive cell divisions and exit the cell cycle as development progresses, a process virtually completed by the end of the first week of postnatal life [9]. The CM compartment enlarges thereafter by hypertrophy that in rodents coincides with binucleation of myocytes [9,10].

Several markers have been individually used to identify CMs (signal regulatory protein alpha [SIRPα], vascular cell adhesion molecule 1 [VCAM-1], Caveolin-3 [Cav3] [11–13]) and their putative adult PRGs (stem cells antigen 1 [Sca-1] [14,15], proto-oncogene receptor tyrosine kinase [c-kit] [16]), FBs (discoidin domain receptor family member 2 [Ddr2], thymus cell antigen 1 [Thy1] [17,18]), SMCs (platelet derived growth factor receptor beta [PDGFrβ] [19]) and endothelial cells (ECs, platelet/endothelial cell adhesion molecule 1 [PECAM-1] [20]). However, because each of these markers is expressed in other cell types, including circulating and heart resident hematopoietic cells, they do not, when used alone, unambiguously define cardiac PRGs or mature populations.

Myocardium regeneration requires the production of new adult CMs. Consistent with a potential regenerative capacity of the adult heart, CM replacement by expansion, albeit at low rate, has been recently reported, indicating that not all adult CMs are postmitotic cells [21,22]. However, the efforts to unravel mechanisms of neocardiomyogenesis have been hampered by the lack of strategies to identify and prospectively isolate the rare CM capable of turnover in normal and diseased hearts.

To identify different maturation stages of CMs and to follow their development up to adulthood, we analyzed the phenotype of all cells in the developing mouse heart. Multiparametric flow cytometry combined with single-cell multiplex quantitative real time polymerase chain reaction (qRT-PCR) of purified cell subsets allows the identification of distinct cell types and the definition of their lineage affiliation. Heat stable antigen (HSA)/cluster of differentiation 24 (CD24) expression is consistently associated with the CM lineage and, combined with other surface proteins, unraveled coexisting subsets of CMs at different stages of maturation. The most immature population expresses HSA, activated leukocyte cell adhesion molecule (ALCAM), melanoma cell adhesion molecule (MCAM), and troponin T (Tnnt), but not Cav3, and is capable of integrating heart tissue after transplantation. The progressive loss of those surface markers contemporaneous with the expression of Cav3 and binucleation identifies mature CMs. Importantly, HSA^+^, but not HSA^−^Cav3^+^ CM, isolated throughout development and up to postnatal day (P) 7 actively proliferate and spontaneously acquire contractile properties in vitro. Isolated from E15 or from P1, they engraft heart tissue transplanted in the ear pinna of adult mice more efficiently than Cav3^+^ CMs. HSA^+^ CMs transcriptional profile resembles that of Cav3^+^ cells, although they express lower levels of transcripts for *Gjc1*, *Arnt*, *Vim*, *Actnb*, and *Casq1* and, in contrast to their Cav3^+^ counterparts, do not express extracellular matrix transcripts, such as *Col3a1*. This population that likely corresponds to cells with a stochastic delayed maturation program persists, albeit at low frequencies, in the adult heart and responds to myocardial infarction (MI) by increasing in numbers.

## Results

### Phenotype assignment to the cellular components in the fetal heart

**Fig 1:**
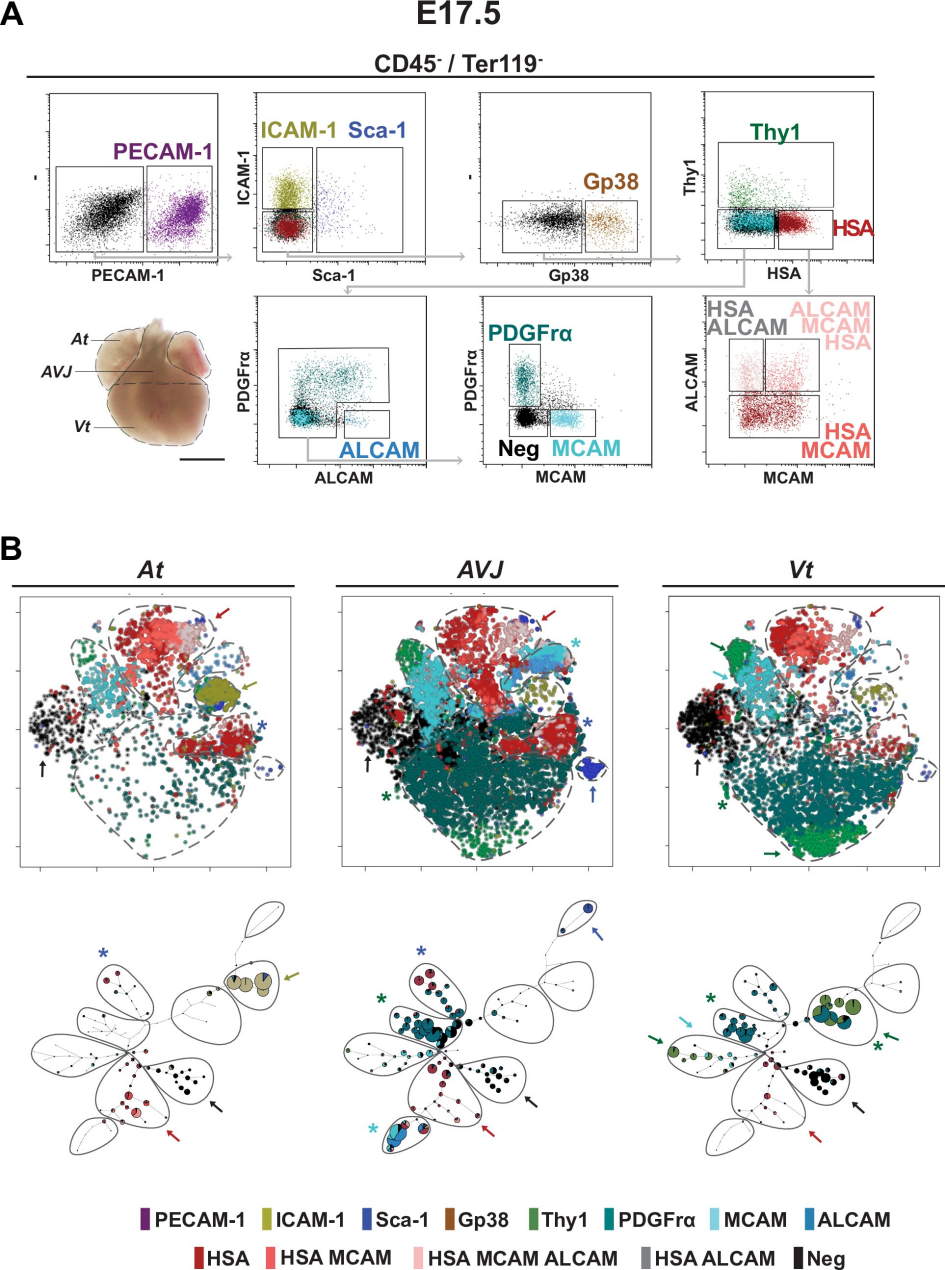
Cell subsets in the fetal heart. (A) Cardiac regions in E 17.5 fetal heart: At, GV-AVJ, and Vt. Scale bar: 1 mm. Flow cytometry dot plots of At CD45^−^Ter119^−^ cells showing the expression of PECAM-1, ICAM-1, Sca-1, Gp38, Thy1, HSA, PDGFrα, ALCAM, and MCAM (*n* = 10). (B) t-SNE (upper graphs) and spanning tree (lower graphs) analyses applied to the populations defined in (A). Each point represents a cell, and colors represent surface signatures (see S1 Fig). Marker expression is represented by a color code: red arrows, HSA, MCAM, and ALCAM; cyan arrows, MCAM; green asterisks, PDGFrα; olive arrows, ICAM-1; green arrows, Thy1; blue asterisks, HSA; blue arrow, Sca-1; cyan asterisk, ALCAM; and black arrows, neg. Colors correspond to populations in (A). The number of dots denotes relative size of the populations. Multiple colors in the same node represent coexpression. ALCAM, activated leukocyte cell adhesion molecule; At, atria; CD45, cluster of differentiation 45; E, embryonic day; Gp38, glycoprotein 38; GV-AVJ, great vessels-atrioventricular junction; HSA, heat stable antigen; ICAM-1, intercellular adhesion molecule 1; MCAM, melanoma cell adhesion molecule; neg, negative for all analyzed markers; PDGFrα, platelet derived growth factor receptor alpha; PECAM-1, platelet/endothelial cell adhesion molecule 1; Sca-1, stem cells antigen 1; Ter119, lymphocyte antigen 76 clone TER-119; Thy1, thymus cell antigen 1; t-SNE, t-distributed stochastic neighbor embedding; Vt, ventricles.

To resolve the phenotype of the cellular components of the developing heart, we screened by flow cytometry cell suspensions isolated from the 3 heart regions (i.e., At, GV-AVJ—containing the connection of the 4 cavities, the great vessels and the valves—and Vt; Fig 1A) for the expression and relative abundance of 30 surface proteins. The analysis was performed in E 17.5 hearts that have similar structure and cellular components to those observed in the adult. We selected 11 antibodies recognizing surface proteins from which HSA (CD24) had not been previously associated with cardiac cells. Following a sequential gating strategy (Fig 1A and S1 Fig), we identified 13 distinct cell populations, after exclusion of hematopoietic (CD45^+^Ter119^+^) cells. Multiparametric analysis of the flow cytometry data by nonlinear dimensionality reduction algorithms (Fig 1B) created maps in which cells clustered together according to phenotype (t-distributed stochastic neighbor embedding [t-SNE], upper graphs) or were organized into hierarchies of related phenotypes (spanning tree, lower graphs). Intercellular adhesion molecule 1 (ICAM-1)^+^ cells (olive arrow) were predominant in the At, Sca-1^+^ (blue arrow), and ALCAM^+^ (cyan asterisk) in the GV-AVJ and Thy1^+^ (green arrow) in the Vt. The remaining subsets were represented in all 3 regions, although at different frequencies (Fig 1B).

**Fig 2:**
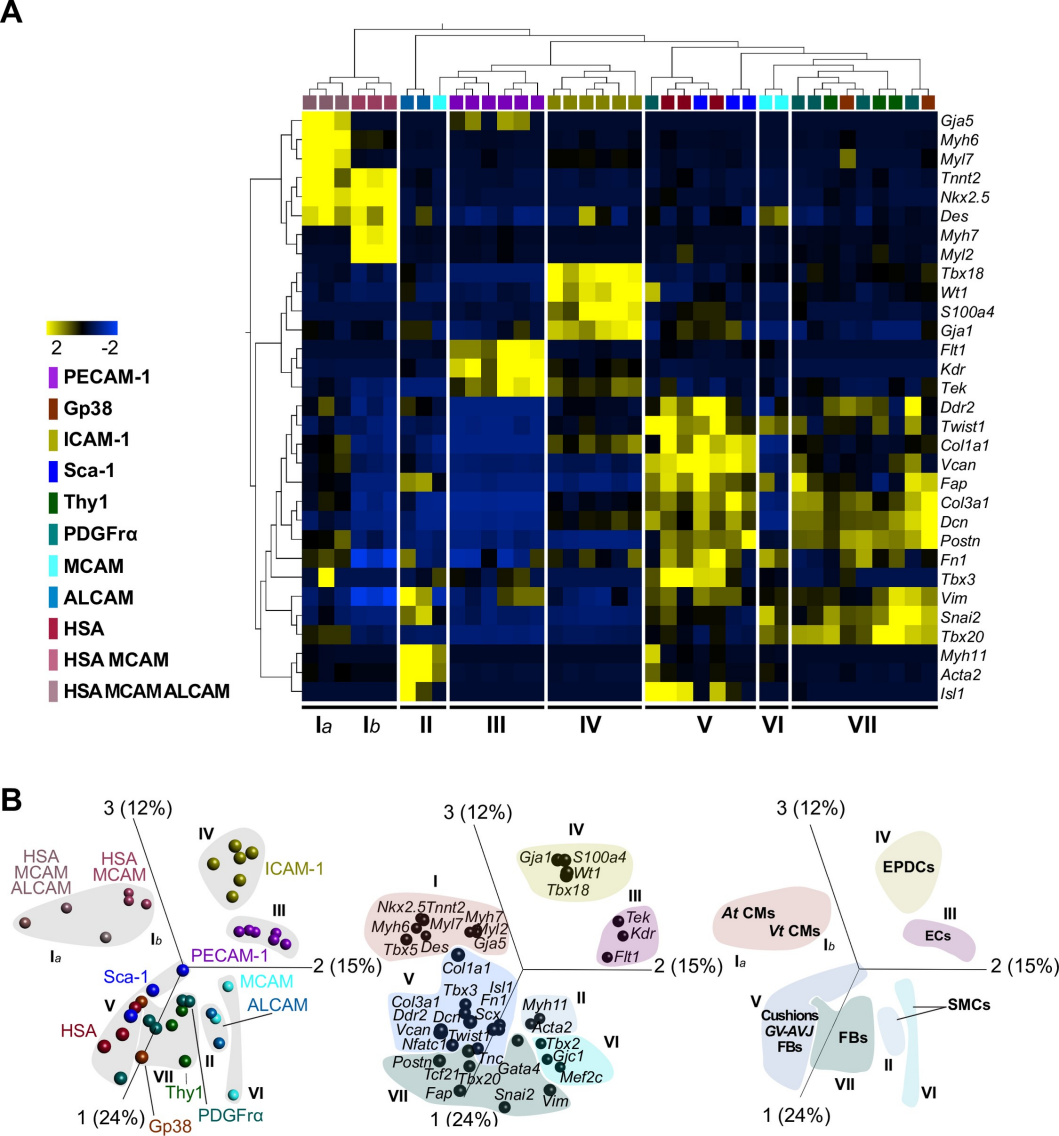
Transcriptional profiles assign cardiac lineages to phenotypes. (A) Unsupervised hierarchical clustering of multiplex qRT-PCR data (20 sorted cells, *n* = 3) in E 17.5 hearts. Differential expressed genes among clusters assign a cell type to each surface signature. Statistical significance was determined using two-way ANOVA; *p* = 0.007, *q* = 0.01 (Table 1). (B) PCA of the transcriptional profile in (A) clustered by surface phenotype (left graph), by gene expression (middle graph), or by cardiac cell type defined by gene expression (right graph). Cluster Ia, At CMs; Cluster Ib, Vt CMs; Cluster II and VI, SMCs; Cluster III, ECs; Cluster IV, EPDCs; Cluster V, Cushions GV-AVJ FBs; and Cluster VII, FBs. The underlying data in (A) and (B) can be found within S1 Data. ALCAM, activated leukocyte cell adhesion molecule; At, atria; CM, cardiomyocyte; E, embryonic day; EC, endothelial cell; EPDC, epicardial-derived cell; FB, fibroblast; Gp38, glycoprotein 38; GV-AVJ, great vessels and atrioventricular junction; HSA, heat stable antigen; ICAM-1, intercellular adhesion molecule 1; MCAM, melanoma cell adhesion molecule; PCA, principal component analysis; PDGFrα, platelet derived growth factor receptor alpha; PECAM-1, platelet/endothelial cell adhesion molecule 1; qRT-PCR, quantitative real time polymerase chain reaction; Sca-1, stem cells antigen 1; SMC, smooth muscle cell; Thy1, thymus cell antigen 1; Vt, ventricle.

**Fig 3:**
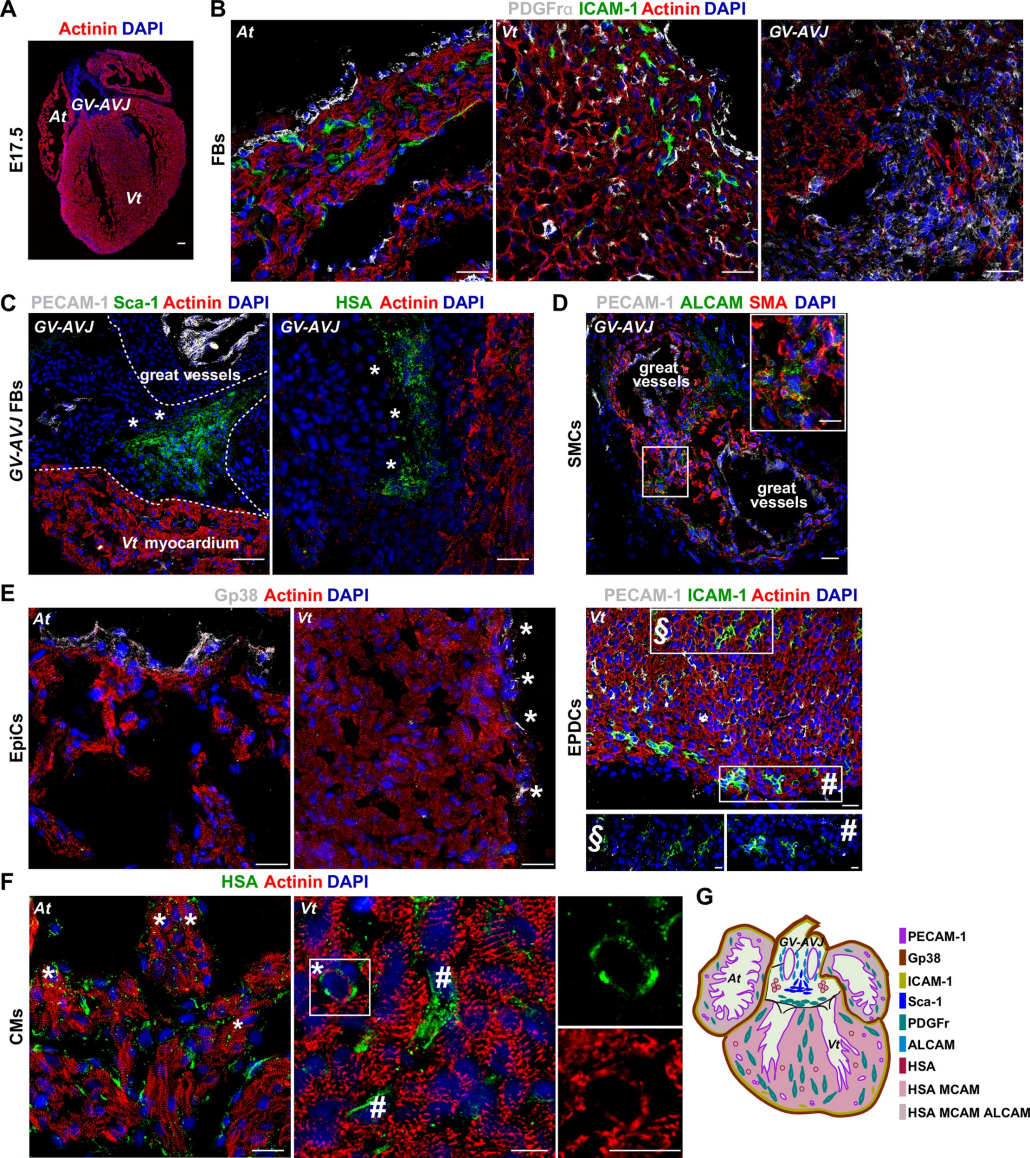
Spatial distribution of cardiac populations. (A) Coronal view of E 17.5 heart section: actinin (red), nuclear content (DAPI, blue). Scale bar: 50 μm. (B) FBs (PDGFrα^+^ cells). (C) GV-AVJ FBs (*, Sca-1^+^PECAM-1^−^ or HSA^+^Actinin^−^ cells). (D) SMCs (ALCAM^+^SMA^+^ cells, inset). (E) EpiCs (2 left panels, Gp38^+^ cells, *); EPDCs (right panel, ICAM-1^+^ PECAM-1^−^, inset #; ECs, ICAM-1^+^PECAM-1^+^ cells, inset §). (F) CMs (HSA^+^Actinin^+^, *; stromal cells, HSA^+^Actinin^−^ cells in the Vt #). Scale bar: 20 μm, insets: 10 μm (3 different sections of each region, *n* = 3). (G) Fetal heart representation with the spatial distribution of the newly defined cardiac populations. ALCAM, activated leukocyte cell adhesion molecule; At, atria; CM, cardiomyocyte; E, embryonic day; EC, endothelial cell; EPDC, epicardial-derived cell; EpiC, epicardial cell; FB, fibroblast; Gp38, glycoprotein 38; GV-AVJ, great vessels and atrioventricular junction; HSA, heat stable antigen; ICAM-1, intercellular adhesion molecule 1; PDGFrα, platelet derived growth factor receptor alpha; PECAM-1, platelet/endothelial cell adhesion molecule 1; Sca-1, stem cells antigen 1; SMA, smooth muscle actin; SMC, smooth muscle cell; Vt, ventricle.

To determine the cell identity of the newly defined populations, we analyzed their transcriptional profiles and anatomical distribution. The expression levels of 31 transcripts affiliated to different cardiac lineages were analyzed in purified cells (20 cells) from each subset. Unsupervised hierarchical clustering and principal component analysis (PCA) grouped the subsets in 7 clusters (Fig 2A and 2B and Table 1). We observed a strong correlation between the clustering by the transcriptional profiles and the subset definition using the cell surface markers, highlighting the validity and the robustness of our approach. Cluster I encompassed CMs identified by the expression of *Nkx2-5*, *Tnnt2*, and *Des*. At CMs (Cluster Ia) expressed At-specific myosin, *Myl7* and *Myh6*, and were phenotypically characterized by the coexpression of HSA, MCAM, and ALCAM. Vt CMs (Cluster Ib) expressing the Vt myosin *Myl2* and *Myh7*, expressed HSA and MCAM but not ALCAM (Fig 2A and 2B). We also analyzed the expression and distribution of the identified proteins in situ (Fig 3A). HSA^+^ CMs identified by the characteristic striated actinin pattern were found in both chambers (more frequent in the At than in the Vt; Fig 3F). In Cluster II, cells expressed *Acta2* together with *Myh11*—indicative of their SMC affiliation—and corresponded to ALCAM^+^ GV-AVJ cells found in the wall of the great vessels, coexpressing smooth muscle actin (SMA) protein (Figs 2A, 2B and 3D). Cluster III cells expressed *Kdr*, *Flk1*, and *Tek* and comprised PECAM-1^+^ ECs and EndoCs (Fig 2A and 2B), delineating the blood vessels (Fig 3C and 3D) and the inner surface of the myocardium (EndoC). Cluster IV cells expressed *Wt1*, *Tbx18*, *S100a4*, and *Gja1*, were identified as ICAM-1^+^ (Fig 2A and 2B), and were observed in the subepicardial region of both chambers (Fig 3B and 3E [inset #]), supporting their EPDC identity. Cluster V combined cells exhibiting a FB transcriptional profile (*Col1a1*, *Col3a1*, *Dcn*, *Twist1*, *Ddr2*, *Vcan*, and *Fn1*) with the expression of GV-AVJ–specific genes *Tbx3* together with *Isl1*, revealing a Sca-1 or HSA phenotype (Fig 2A and 2B). Sca-1^+^ (PECAM-1^−^) cells were detected at the insertion of great vessels (Fig 3C, *), whereas HSA^+^ cells (actinin^−^) were observed in the EndoC cushion mesenchyme (Fig 3C, *). Cluster VI corresponded to MCAM^+^ cells expressing the SMC-associated transcript *Acta2* together with *Tbx20*, *Snai2*, *Vim*, *Fn*, and *Fap* (Fig 2A and 2B). Finally, in Cluster VII, glycoprotein 38 (Gp38)^+^, PDGFrα^+^, and Thy1^+^ cells were grouped by the expression of a different combination of FB-related transcripts (*Ddr2*, *Col3a1*, *Dcn*, *Postn*, *Fn*, *Vim*, *Snai2*, *Tbx20*; Fig 2A and 2B). PDGFrα expression was associated with FBs in the myocardium (Fig 3B), whereas Gp38^+^ cells were detected in the epicardium (Fig 3E, *).

**Table 1:** Summary of the E 17.5 cardiac lineage properties.

E 17.5					
Cluster	Genes	Cell type	Location		Cell-surface phenotype
			Anatomical	Histological	
Ia	Nkx2-5***, Tnnt2***, Des***, *Myl7*, *Myh6*	At CMs	At	Widespread in myocardium	HSA^+^MCAM^+^ ALCAM^+^
Ib	Nkx2-5***, Tnnt2***, Des***, *Myl2*, *Myh7*	Vt CMs	Vt	Small foci in myocardium	HSA^+^MCAM^+^
II	Acta2***, Myh11***	SMCs	GV-AVJ	Great vessels wall	ALCAM^+^
III	Kdr***, Flt1***, Tek***	ECs	At, GV-AVJ, Vt	Endothelium and endocardium	PECAM-1^+^
IV	Tbx18***, Wt1***, S100a4***, Gja1***	EPDCs	At, GV-AVJ, Vt	Sub-EpiC zone	ICAM-1^+^
V	Twist1***, Tbx3***, Vcan***, Fap***, Vim***, Isl1***	GV-AVJ and EndoC cushions FBs	GV-AVJ	EndoC cushions	HSA^+^
				Great vessels insertion	Sca-1^+^
VI	*Tbx20*, *Snai2*, *Vim*, *Fn*, *Fap*	SMCs	GV-AVJ, Vt	-	MCAM^+^
VII	*Ddr2*, *Col3a1*, *Dcn*, *Postn*, *Fn1*, *Vim*, *Snai2*, *Tbx20*	EpiCs	At, GV-AVJ, Vt	Epicardium	Gp38^+^
		FBs	GV-AVJ, Vt	-	Thy1^+^
			At, GV-AVJ, Vt	Interstitium	PDGFrα^+^

*** *p* = 0.000435; *q* = 0.0022 by one-way ANOVA

**Abbreviations:** ALCAM, activated leukocyte cell adhesion molecule; At, atria; CM, cardiomyocyte; E, embryonic day; EC, endothelial cell; EndoC, endocardial cell; EPDC, epicardial-derived cell; EpiC, epicardial cell; FB, fibroblast; Gp38, glycoprotein 38; GV-AVJ, great vessels and atrioventricular junction; HSA, heat stable antigen; MCAM, melanoma cell adhesion molecule; PECAM-1, platelet/endothelial cell adhesion molecule 1; PDGFrα, platelet derived growth factor receptor alpha; Sca-1, stem cells antigen 1; SMC, smooth muscle cell; Thy1, thymus cell antigen 1; Tnnt, troponin T; Vt, ventricle

To probe heterogeneity of the cardiac subsets, we carried out single-cell transcriptional analyses (S2A and S2B Fig). Cell sorting was performed using the index-sorting tool that records, for each cell, the levels of expression of each phenotypic parameter (S2C Fig). Both the heat map and the PCA analysis indicated that the phenotypically defined populations homogeneously coexpressed the analyzed transcripts, underscoring the strength of this combined approach (S2A and S2B Fig).

The ensemble of these results validated the phenotyping strategy (Fig 1 and Table 1) that allowed the identification (Figs 2A, 2B and 3G) and the prospective isolation of the major cardiac cell subsets in the fetal heart.

### Different stages of CM maturation coexist during development

**Fig 4:**
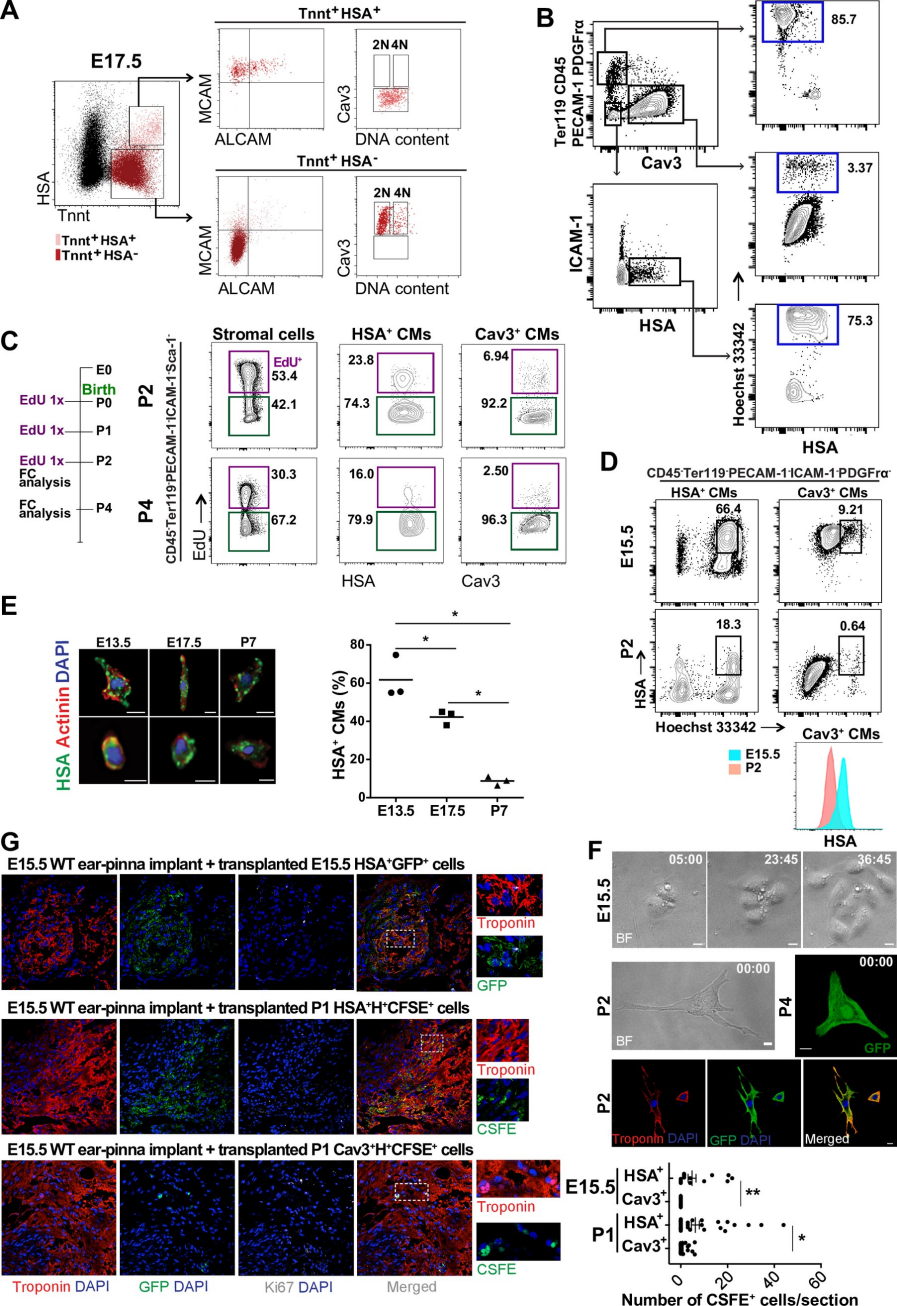
Expression of HSA and Cav3 identify 2 CM subsets. (A) E 17.5 whole-heart suspensions (*n* = 2). HSA^+^MCAM^+^ALCAM^+/−^Cav3^−^ (light pink, upper panels) and HSA^−^MCAM^−^ALCAM^−^Cav3^+^ (red, lower panels) analyzed for DNA content. (B) Flow cytometry profiles of P5 whole-heart suspensions (the great vessels were dissected out) stained with Cav3, HSA, ICAM-1, and Hoechst 33342, after exclusion of CD45-, Ter119-, PECAM-1–, and PDGFrα-expressing cells. Gating strategy and Hoechst 33342 (H^+^) expression in PECAM-1^+^, PDGFrα^+^, and CD45^+^ cells (upper right panel), Cav3^+^ CMs (middle right panel), and HSA^+^ CMs (lower right panel). (C) EdU incorporation in CD45^−^Ter119^−^PECAM-1^−^ICAM-1^−^Sca-1^−^, PDGFRα^+^ stromal cells (left), HSA^+^ CMs (middle), and Cav3^+^ CMs (right) in P2 (top) and P4 (bottom) hearts (*n* = 2). (D) HSA expression levels in E 15.5 HSA^+^H^+^ CMs (upper left), Cav3^+^H^+^ CMs (upper right), in P2 HSA^+^H^+^ CMs (lower left), and Cav3^+^H^+^ CMs (lower right). Histogram compares the HSA expression level of Cav3^+^H^+^ CMs during maturation (E 15.5 and P2). (E) HSA expression in CMs (Actinin^+^) by imaging flow cytometry. Scale bar: 10 μm. Frequency of HSA^+^ CMs at E 13.5, E 17.5, and P7 (*n* = 3; 59,397; 67,306; and 7,088 total cells analyzed for E 13.5, E 17.5, and P7, respectively). (F) Cultured HSA^+^ E 15.5 CMs (time lapse, hour:minute, upper panels, MS1, MS2), HSA^+^ P2 and P4 CMs (middle panels and MS3, MS4). Scale bar: 10 μm (*n* = 3). Immunofluorescence of cultured (72 hours) P2 CMs. Tnnt (in red) and GFP (Ub–GFP cells, lower panels). (G) Immunofluorescence of cardiac implants transplanted with Ub–GFP^+^ E 15.5 HSA^+^ CMs (upper panel, *n* = 3 biologic replicates, with 4 technical replicates in each experiment), WT P1 HSA^+^H^+^CFSE^+^ (middle panels), or Cav3^+^H^+^CFSE^+^ (lower panels, *n* = 2 biologic replicates, with 2 technical replicates in each experiment, upper panel). Cardiac Tnnt (red), GFP or CFSE (green, donor cells), Ki67 (white), and DAPI (nuclei, blue). Scale bar: 50 μm. Insets show higher magnification of the region within the dashed white rectangle (right panels). *p < 0.05. The underlying data in (E) and (G) can be found within S2 Data. ALCAM, activated leukocyte cell adhesion molecule; Cav3, Caveolin-3; CD45, cluster of differentiation 45; CFSE, carboxyfluorescein succinimidyl ester; CM, cardiomyocyte; E, embryonic day; EdU, 5-ethynyl-2´-deoxyuridine; FC, flow cytometry; GFP, green fluorescent protein; HSA, heat stable antigen; ICAM-1, intercellular adhesion molecule 1; Ki67, Kiel clone 67; MCAM, melanoma cell adhesion molecule; P, postnatal day; PDGFrα, platelet derived growth factor receptor alpha; PECAM-1, platelet/endothelial cell adhesion molecule 1; Sca-1, stem cells antigen 1; Ter119, lymphocyte antigen 76 clone TER-119; Tnnt, troponin T; Ub–GFP, Ubiquitin–GFP; Vt, ventricle; WT, wild type.

A CM transcriptional profile (*Nkx2-5*, *Tnnt2*, and *Des*) was associated with membrane HSA expression in different, but related, cell populations (Fig 2) isolated from both heart chambers. To understand the kinetics of the newly defined populations along heart morphogenesis, we performed a similar analysis at earlier developmental stages (E 9.5 and E 13.5; S3A and S3D Fig). HSA^+^MCAM^+^ALCAM^+^ (triple positive) CMs were detected in all analyzed embryonic stages. However, by E 17.5, most Vt, but not At, HSA^+^ CMs had lost ALCAM expression (S3A Fig). HSA^+^MCAM^+^(ALCAM^−^) and HSA^+^(MCAM^−^ALCAM^−^) CMs were more frequent in the E 17.5 Vt (S3A Fig, bottom right plot), and triple negative CMs (HSA^−^MCAM^−^ALCAM^−^), initially detected in the E 13.5 Vt, increased to comprise most CMs at E 17.5 (Fig 4A and S3D Fig). Cell cycle analysis showed that E 9.5 HSA^+^ cells, with a transcriptional profile of CM PRGs (S3A Fig), were highly proliferative (63.7% in gap 1 phase [G_1_] and 15.9% in synthesis phase/gap 2 phase-mitosis [S/G_2_-M]; S3B Fig). As development proceeded, the frequency of HSA^+^ cells decreased, and they also divided less (14.4% versus 7.66% were in S/G_2_-M at E 13.5 and E 17.5, respectively; S3B Fig).

To investigate whether the phenotypically distinct CM subsets corresponded to different stages of maturation, we included Tnnt [23] and Cav3 [12] in our analysis. Surface Cav3 expression is detected in the adult but is undetectable in most immature CMs [12], whereas Tnnt is widely expressed in the CM lineage [23,24]. The transcriptional profile of the HSA^+^ CMs along time (E 9.5, E 13.5, and E 17.5) revealed that Cav3 starts to be transcribed at E 9.5 (S2D Fig), and the first Cav3^+^ cells detected by protein staining still express high levels of HSA (Fig 4D), suggesting a lineage relationship between the 2 CM subsets. These results highlight the progressive loss of the surface markers ALCAM, MCAM, and HSA coinciding with the transcriptional expression of Cav3 at E 13.5, whereas at E 17.5 the majority of CMs (Tnnt^+^) were HSA^−^MCAM^−^ALCAM^−^Cav3^+^ (Fig 4A). During maturation, CMs lose proliferative capacity and suffer morphologic alterations leading to increased sarcomere complexity, bigger cell size, and binucleation [9]. Cell-cycle analysis of E 17.5 CM subsets (HSA^+^ and Cav3^+^) showed that HSA^+^ cells were still proliferative (At: 24.4% and 20.5%; and Vt: 35.5% and 25.3%, in G_1_ and S/G_2_-M, respectively; S3C Fig) not only at the end of gestation but also after birth (P2: 21.9% and 22.7% in G_1_ and S/G_2_-M, respectively; and P5: 10.4% and 31.1% in G_1_ and S/G_2_-M, respectively; S3E Fig). By contrast, Cav3^+^CMs were largely out of cycle (At: 69.4% and Vt: 78% in G_0_; S3C Fig). Of note, in P15 hearts, the few HSA^+^ CMs detected, although not actively proliferating, showed a consistent proportion of cells in the G_1_ stage of the cell cycle (S3E Fig). Stromal subsets were highly proliferative (At: 36.6% and 11.2%; Vt: 27.6% and 11.3%, respectively, in G_1_ and S/G_2_-M; S3C Fig). Binucleated cells (4N DNA) were observed in the Tnnt^+^HSA^−^Cav3^+^ CM population, whereas Tnnt^+^HSA^+^Cav3^−^ CMs exhibited a continuum increase in DNA content, consistent with nucleic acid synthesis (Fig 4A, S3C and S3E Fig). For the first time, to our knowledge, binucleated CMs were found during development and can be isolated using HSA^−^Cav3^+^ as a surface signature.

Our results demonstrate the coexistence of 4 stages of CM maturation along development (E 9.5–E 17.5). Immature HSA^+^MCAM^+^ALCAM^+^ CMs are the dominant subset in the At. The expression of these markers is progressively lost such that at E 17.5 only a small fraction of Vt CMs is still HSA^+^MCAM^+^ALCAM^−^, whereas the majority of CMs are negative for all markers, display surface Cav3, and have initiated binucleation (Fig 4A, S3C Fig). At CMs retained the expression of the 3 markers until later embryonic stages than their Vt counterparts (S3A Fig), consistent with the well-documented differences in CM maturation of the 2 chambers [25].

Because HSA is the last surface protein to be lost before the acquisition of Cav3, we used these 2 markers to discriminate immature and mature CMs, respectively. Interestingly, HSA and Cav3 expression define 2 CM subsets with different proliferative capacity, even after birth. Three 5-ethynyl-2´-deoxyuridine (EdU) injections (P0, P1, and P2) in the neonates labeled 23% of HSA^+^ CMs detected at P2 and 16% at P4, indicating that this CM subset maintains proliferative activity after birth (Fig 4C). By contrast, only 7% of Cav3^+^ CMs incorporated EdU, demonstrating a lower contribution of the mature CM subset to postnatal heart growth (Fig 4C). Other non-CM (stromal) cells showed more than 50% EdU incorporation, compatible with their high proliferative activity at this stage (Fig 4C). The reduced frequency of Cav3^+^ CMs at P7 detected by flow cytometry (S3D Fig, contour plots) reflects the sensitivity of postnatal CMs to enzymatic digestion. Compatible with this possibility, although most Cav3^+^ CMs did not incorporate DNA intercalating agents, such as propidium iodide (PI), used in the flow cytometry analysis, they also did not stain (>4%) with Hoechst 33342 that detects DNA in live cells (Fig 4B). By contrast, more than 70% of HSA^+^ CMs showed levels of DNA after Hoechst 33342 staining similar to live ECs (PECAM-1^+^PI^−^) or stromal cells (PDGFrα^+^PI^−^). Consistently, we found that Hoechst positive and negative cells yielded a more than 30-fold difference in the amplified cDNA for *Hprt*, although with similar relative amounts of *Tnnt2* transcripts (S4D Fig). These results indicate that different subsets of CM have different sensitivity to enzymatic digestion that bias the analysis of postnatal CMs in cell suspension.

To overcome the impaired cell integrity of postnatal CMs, we performed a similar analysis in cells fixed before dissociation. Kinetics of HSA and Cav3 expression showed that the decrease of the former parallels an increase of the latter (S4C Fig). At E 13.5, we detected HSA^+^ (27.3% ± 3.3%), HSA^+^Cav3^+^ (10.3% ± 1.2%), and a few cells expressing Cav3 (10.3% ± 2.6%). At E 17.5, the majority of the cells expressed Cav3 (44.8% ± 6.3%); a fraction of them were binucleated (4.1% ± 0.9%) and coexisted with either HSA^+^ (4.9% ± 1.5%) or HSA^+^Cav3^+^ (3.9% ± 1.9%). After birth (P7), the majority of cells expressed Cav3 (53.1% ± 5.1%) and presented 2 (2 nuclei, 38.7% ± 2.5%, S4C Fig) or more nuclei (>2 nuclei, 2.5% ± 0%, S4C Fig), whereas we failed to detect multinucleated HSA^+^ cells (S4C Fig). CMs fixed prior to isolation were also analyzed by imaging flow cytometry, evidencing decreased percentage of immature HSA^+^ CMs along development (57.8% ± 11.3% at E 13.5 and 38.3% ± 3.8% at E 17.5), with a pronounced decline after birth (4.8% ± 2.3% at P7; Fig 4E). Importantly, HSA expression was only observed in mononucleated CMs, further associating its expression with an immature phenotype (Fig 4E).

To confirm that HSA discriminates immature CM, purified HSA^+^ cells isolated from E 15.5, P2, and P4 hearts were cultured for up to 1 week. E 15.5 HSA^+^ cells either divided (approximately 20%) or were contractile (approximately 75%) in culture, whereas no proliferation was observed in P2 or P4 cardiac cells, probably due to the rapid differentiation in culture (Fig 4F, S6A Fig, MS1 and MS2). Seeded P2 and P4 cells adhered and were contractile at a frequency ranging from 1:30 to 1:40 (S6B Fig); by contrast, Cav3^+^ cells, irrespective of the stage at which they were isolated, did not adhere to gelatin-coated plates (more than 10,000 cells analyzed) and were not viable after 3 days in culture (S6B Fig). All P2 and P4 adherent cells showed contractility (S6B Fig, S3 and MS4). The nonorganized pattern of Tnnt observed on contractile HSA^+^ CMs in culture reflects their immaturity (Fig 4F) and is a feature also observed during development (Figs 3F and 4E, actinin staining).

To show that immature CMs have the capacity to integrate cardiac tissue in vivo, we used a previously described experimental model [26], in which cardiac cells are transplanted in an ectopic heart tissue implanted in the ear pinna of adult mice (S8A Fig). Viable functional implants were identified by autonomous beating ascertained by visual inspection and by the presence of Kiel clone 67 (Ki67)^+^ mitotic cells (S8C Fig). A first set of experiments was performed with E 15.5 HSA^+^ CMs from Ubiquitin–GFP (Ub–GFP) mice to allow the identification of the donor cells (Fig 4G, upper panels and S8C Fig). Because most Cav3^+^ cells were not viable (H^−^) and therefore did not express GFP in the Ub–GFP model, we isolated Cav3^+^ H^+^ cells from wild-type (WT) mice and labeled with carboxyfluorescein succinimidyl ester (CFSE) dye that binds to cytoplasmic proteins, resists to fixation, and can be detected after several rounds of division [27]. Seven days after WT E 15.5 Vt tissue implantation, we transplanted either E 15.5 HSA^+^GFP^+^ CMs from Ub–GFP mice (upper panels) or from WT mice E 15.5 or P1 HSA^+^ and Cav3^+^ CMs and labeled with CFSE (middle and lower panels, Fig 4G). One week after cell transplantation, the implants injected with HSA^+^ CMs displayed regions of GFP^+^ or CFSE^+^ cells with the characteristic striated pattern of myocytes that also coexpressed Tnnt (Fig 4G). A quantification of the number of donor cells detected in the explants (cells per section) indicated HSA^+^ CMs from P1 hearts integrate the grafted tissue more efficiently than P1 Cav3^+^ CMs and that no significant difference is found between E 15.5 and P1 HSA^+^ CMs. Together, these results demonstrated the better efficiency of immature HSA^+^ CMs compared with Cav3^+^ CMs to engraft cardiac tissue and maintain viability for up to 1 week (Fig 4G and S8 Fig).

**Fig 5:**
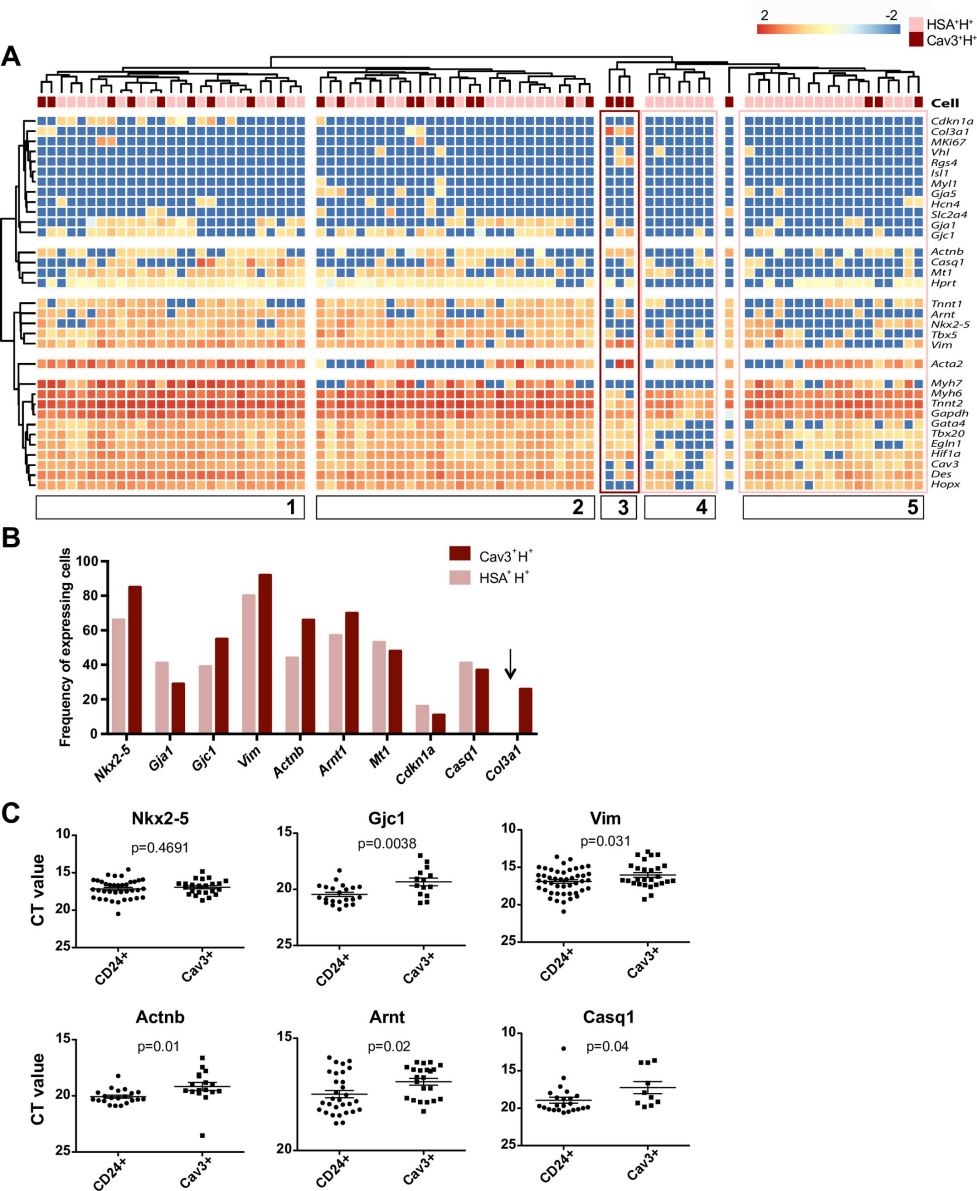
Immature HSA^+^ CMs exhibit lower expression of cardiac structural transcripts than mature Cav3^+^ CMs. (A) Unsupervised hierarchical clusters obtained from a single-cell multiplex qRT-PCR analysis of P1 HSA^+^H^+^ and Cav3^+^H^+^ cells (84 analyzed single cells). Only cells expressing at least 1 housekeeping gene were considered. Five different clusters were defined: 2 containing a mixture of both CM subsets, 1 with only Cav3^+^ cells (dark pink square), and 2 with HSA^+^ cells (light pink squares). (B) Frequency of cells expressing each of the designated transcripts (chosen by differential expression). The arrow indicates a transcript exclusively found in Cav3^+^ CMs. (C) Ct values of the differentially expressed transcripts in each CM population (inversely proportional to expression levels). The underlying data in (A–C) can be found within S3 Data. Cav3, Caveolin-3; CD24, cluster of differentiation 24; CM, cardiomyocyte; Ct, cycle threshold value; HSA, heat stable antigen; P, postnatal day; qRT-PCR, quantitative real time polymerase chain reaction.

The transcriptional profile of P1 HSA^+^H^+^ and Cav3^+^H^+^ CMs probed for over 40 transcripts (assembled from our previous panel and complemented with transcripts differentially expressed between E 14.5 and P0 [28]) indicated that most cells from both subsets exhibited a similar transcriptional profile (Fig 5A and 5B). This result is compatible with the observation that Cav3^+^H^+^ cells express higher levels of HSA than their H^−^ counterparts, suggesting a recent transition to the Cav3^+^ compartment (Fig 4D). Five major clusters were identified: (i) cluster 1 is mostly composed of HSA^+^H^+^ cells expressing high levels of *Acta2*, *Vim*, *Tbx5*, *Nkx2*.*5*, *Arnt*, *Gjc1*, *Gja1*, and *Cdkn1a*; (ii) cluster 2 has a stronger representation of Cav3^+^H^+^ and differed from cluster 1 by lower expression of *Acta2* and absence of *Cdkn1a* expression; (iii) cluster 3 of Cav3^+^H^+^ cells is characterized by high levels of *Col3a1*, *Actnb*, *Rgs4*, and *Vim* and the absence of *Myh7*; (iv) clusters 4 and 5 are composed of HSA^+^H^+^ cells characterized by low expression of *Actnb*, *Arnt*, *Nkx2*.*5*, *Tbx5*, and *Vim* and differ from each other by differential expression of *Acta2*, *Myh7*, *Tbx20*, and *Egln1*. Overall, HSA^+^ CMs expressed significantly lower levels of transcripts for the structural cardiac proteins *Gjc1*, *Vim*, *Actnb*, *and Casq1*, indicative of their more immature stage (Fig 5C) [9]. No HSA^+^ CMs were shown to express extracellular matrix (ECM) specific transcripts, suggesting that the recently described CM population [28] belongs to the Cav3^+^ subset (Fig 5A and 5B [arrow]). Remarkably, though, all cells isolated on the basis of the unique expression of HSA, and negative for CD45, Ter119, PECAM-1, and PDGFα, consistently expressed high levels of *Tnnt2*, *Myh6*, *Gata4*, and *Des*, demonstrating the relevance of this marker to identify immature CMs in the mouse (Fig 5A).

Overall, our results identify distinct stages of CM maturation along development based on surface-marker expression. We defined 2 major CM subsets: an immature subset of mononucleated cells with proliferative capacity (HSA^+^) that can give rise to functional CMs both in vitro and in vivo and a mature fraction (Cav3^+^) with increased sarcomere complexity, binucleation, and decreased proliferative capacity.

#### Immature CMs persist in adulthood and increase after injury

**Fig 6:**
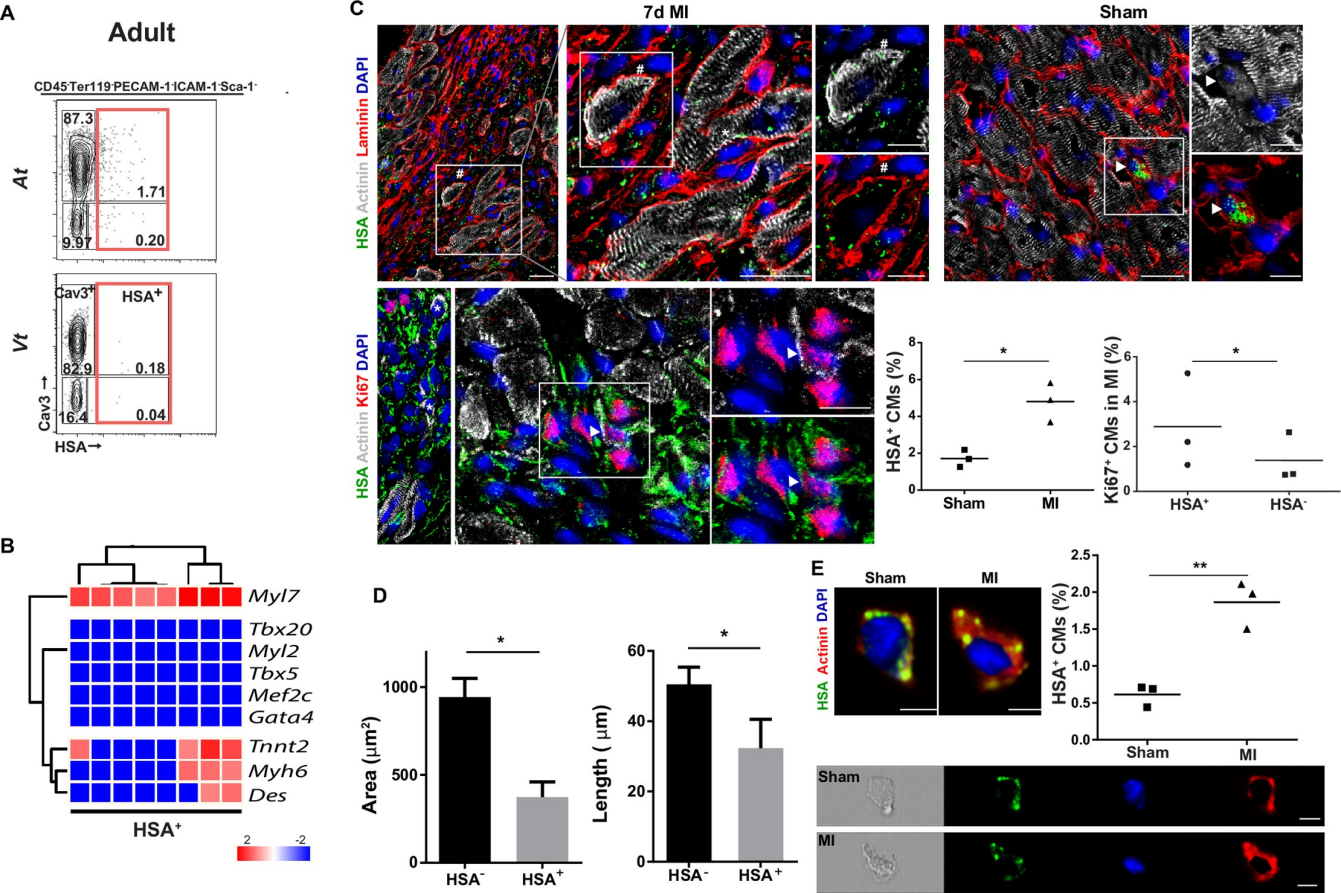
Immature CMs in the adult heart respond to injury. (A) Flow cytometry frequency and (B) gene expression of adult HSA^+^ single cells (*n* = 2). (C) Immunofluorescence of the peri-infarcted myocardium of MI (left panels) or sham-operated animals (right panel). HSA^+^ CMs (arrowhead), small round (*), or large striated HSA^+^ CMs (#); Ki67^+^HSA^+^ CMs (arrowhead). Frequency of HSA^+^ CMs (Actinin^+^) per heart after MI (49 sections across 3 hearts) and in sham-operated animals (30 sections across 3 hearts; right graph). Frequency of proliferative (Ki67^+^) CMs (Actinin^+^) expressing or not HSA per heart after MI (75 sections across 3 hearts; left graph).*p < 0.05. Scale bar: 20 μm, insets 10 μm. (D) Histogram bar graphs show length and area of HSA^+^ and HSA^−^ CMs (*n* = 3; 9,415 cells), in imaging flow cytometry. (E) Imaging flow cytometry of CMs in MI and sham-operated hearts. Frequency of HSA^+^ CMs (Actinin^+^) isolated from sham-operated (*n* = 3; 43,461 cells analyzed) and MI hearts (*n* = 3; 35,189 cells analyzed). Scale bar: 10 μm. **p < 0.005. The underlying data in (B–E) can be found within S4 Data. At, atria; Cav3, Caveolin-3; CD45, cluster of differentiation 45; CM, cardiomyocyte; HSA, heat stable antigen; ICAM-1, intercellular adhesion molecule 1; Ki67, Kiel clone 67; MI, myocardial infarction; PECAM-1, platelet/endothelial cell adhesion molecule 1; Sca-1, stem cells antigen 1; Vt, ventricle.

To determine whether immature HSA^+^ CMs persist in adulthood, we analyzed adult heart cell suspensions with the antibody panel defined above. In P21 heart cell suspensions, we found 1.76% HSA^+^ CMs that expressed low levels of Cav3 and 30% of which are also H^+^, whereas only 0.8% of Cav3^+^ CMs are H^+^ (S9B Fig). CM-specific transcripts (*Tnnt2*, *Myl7*, and *Myh6*; Fig 6B) were present in adult HSA^+^ cells, more frequent in the At (At: 1.7% and Vt: 0.18%; Fig 6A), but also in HSA^−^ cells expressing surface Cav3 (Fig 6A and S9C Fig). Imaging flow cytometry analysis further confirmed the presence of a discrete subset of HSA^+^Actinin^+^ CMs in adult hearts (0.6% ± 0.33%, *n* = 3) restricted to the subset of mononucleated CMs smaller (in area and length) than HSA^−^ CMs (Fig 6D). The HSA^+^ subset was considered as the most immature adult CMs because they shared cytological and phenotypic properties with embryonic CMs, in culture adhered to gelatin-coated plates, survived for a few days, and expressed Tnnt but failed to divide or to contract (S7B Fig), and the majority also coexpressed the mature marker Cav3 (Fig 6A). Two stromal populations were also discriminated in the adult: (i) a PDGFrα^+^Sca-1^+^Thy1^low^ subset of FBs compatible with a previously described stromal population [29] and (ii) ICAM-1^+^Gp38^+^Thy1^+^ cells, expressing *Gata4*, *Tek*, *Dcn*, *Twist1*, and *Tbx18* and located in the subepicardial region (S9A, S9C and S9D Fig). c-kit expression previously associated with CM PRGs was exclusively found in adult PECAM-1^+^ ECs, and transcripts were also detected in ICAM-1^+^ sub-EpiCs (S9C Fig).

The detection of an immature CM subset in the adult prompted us to investigate its frequency in the diseased heart. We found HSA expression largely circumscribed to the non-CM compartment in sham-operated hearts, although rare HSA^+^Actinin^+^ CMs were also detected (outlined by laminin expression, Fig 6C). Seven days after MI, and in spite of HSA expression associated to the upsurge of the hematopoietic and ECs (S9E Fig), we detected a 3-fold increase in the frequency of HSA^+^ small-round and large-striated mononucleated CMs (# and *, respectively; 4.7% ± 3.6%) compared with sham-operated (1.7% ± 1.1%; Fig 6C). HSA^+^ CMs were found in the peri-infarcted region as shown in the lower magnification image (Fig 6C). Moreover, a small percentage of HSA^+^ CMs (i.e., approximately 1 per section) were in cycle (Ki67 expression) after MI (Fig 6C). A similar increase in HSA^+^ CMs was evidenced after MI by imaging flow cytometry analysis of prefixed cells (1.8% ± 0.3%) compared with sham-operated hearts (0.6% ± 0.15%; Fig 6E).

Our results show that the cell surface signatures defined for the embryonic heart are suitable to isolate an affiliated adult population. Importantly, we identified a small subset of HSA-expressing adult CMs that, like their embryonic counterparts, are mononucleated, express low levels of Cav3, and exhibit a higher probability than Cav3^+^ cells to proliferate and increase in frequency after MI.

## Discussion

The mouse heart is able to regenerate during the first days of life by proliferation of pre-existing CMs. This capacity is lost after 1 week of postnatal life, the time from which wounded tissue is replaced by a nonfunctional scar [30]. The loss of CM mitotic activity has been attributed to the binucleation process and the complete maturation of CMs that occur after birth [10,31]. However, recent reports showed that CM replacement and cell division could occur, although at low rate [21,22], raising the possibility that a subset of CMs in the adult might undergo mitosis. Specific surface markers need to be identified to allow the isolation and characterization of the low frequency of dividing CMs. We show here that, although HSA is not a marker for proliferating cells, alone it identifies a neonatal CM compartment that retains higher proliferative capacity, persists in the adult, and expands after MI. HSA has been associated with proliferating cells in several tissues (e.g., skeletal muscle PRG cells [32,33]), as well as in several cancer types [34]; however, its function is unknown. Although HSA is detected in all CMs in E 9.5 (S5C Fig), the constitutive inactivation of HSA does not appear to have a strong impact in heart development, because these mice are viable and fertile. It has, however, been reported that the fraction of born homozygous pups is lower than predicted, raising a possibility of a low penetrance lethal effect [32].

Expression of *Isl1*, *Gata4*, *Mef2c*, and *Tbx20* is important for CM commitment and initial stages of differentiation [35–37], and accordingly they were used as indicative of the CM lineage. Consistent with previous reports [29], we found some of these transcripts also expressed during the development of stromal cells (SMCs and FBs), suggesting that, in the heart, they participate in the development of different lineages. These findings demonstrate that CMs can only be unambiguously identified by the combined expression of transcription factors, transcripts codifying structural and contractile proteins, and by the absence of stromal- or EC-associated markers.

Several studies identified CM PRGs based on the expression of Sca-1 and c-kit [14–16] cell-surface proteins, which in our work were not found within the CM compartment. c-kit was only expressed in ECs (PECAM-1^+^), as recently shown also by others [38], and in Thy1^+^PDGFrα^+^ FBs. Sca-1 expression was detected in a fraction of ECs and in a population of cells in the atrioventricular canal in the fetal heart and in the interstitium of adult myocardium [15]. Their spatial pattern and transcriptional profile are compatible with an FB lineage affiliation, supported by the description of Sca-1-expressing cells exhibiting a paracrine role in angiogenic stimulation after MI [15,39]. At E 9.5, we found an HSA, ALCAM, and PDGFrα expressing population that was highly proliferative and expressed *Nkx2-5* and *Tnnt2* together with *Isl1* and *Tbx5*, suggesting they represent CM PRGs (S2D Fig) [40, 41]. These cells sharply decrease in frequency, they are only detected in the At after E13.5 of development, and become undetectable after birth, a finding that is not compatible with the persistence of CM PRGs in the postnatal heart.

**Fig 7:**
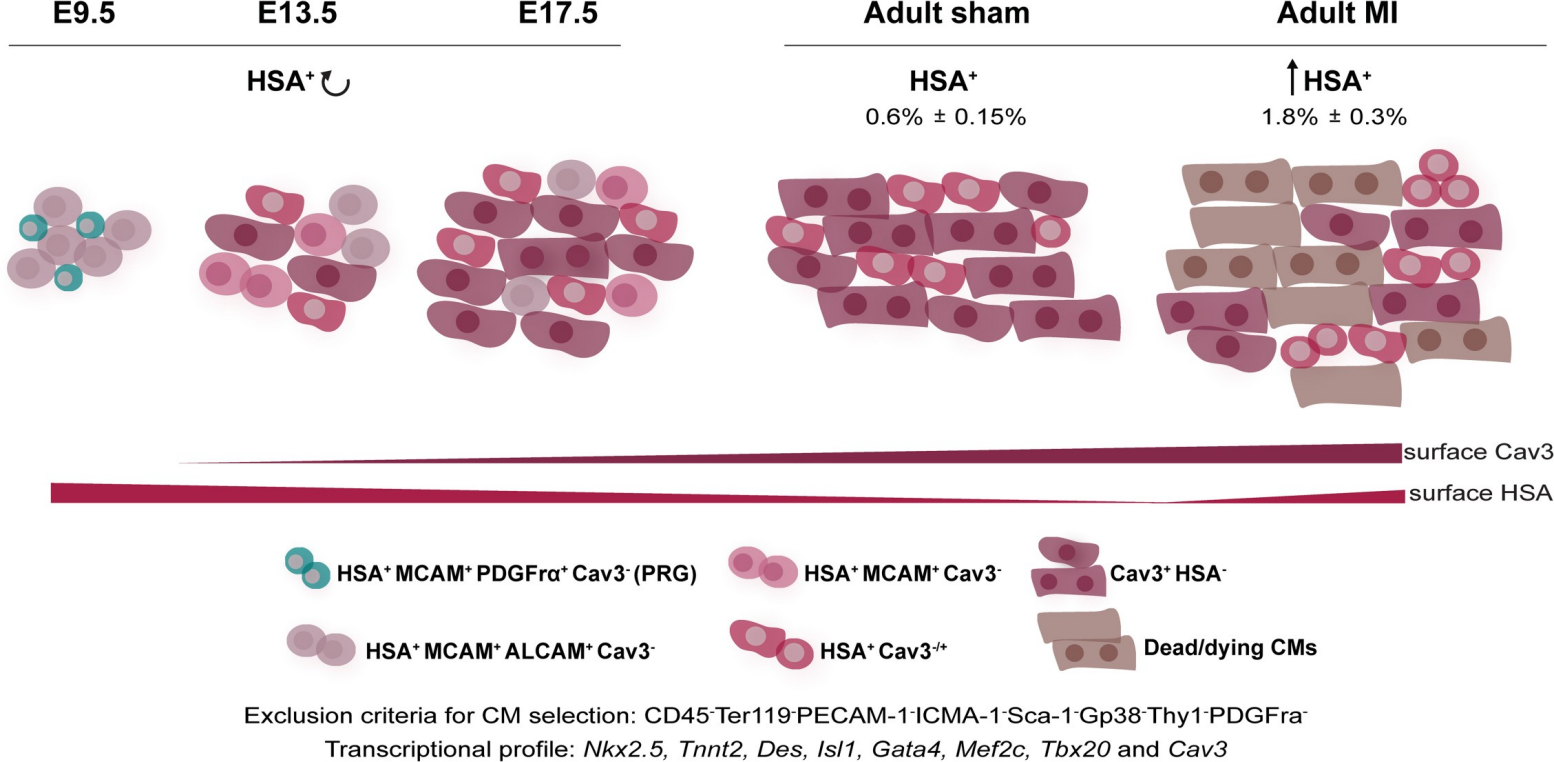
HSA is as a transversal marker of immature CMs. The combined use of surface marker analysis with single-cell transcriptional profiling allowed identifying different subsets of CMs (expressing *Nkx2*.*5*, *TnnT2*, *Des*, *Isl1*, *Gata4*, *Mef2c*, and *Tbx20*) in the murine heart. Less mature CMs (Tnnt^+^Cav3^−^) express HSA, MCAM, and ALCAM and progressively lose these markers, maintaining the expression of HSA. Cav3 transcripts are, however, detected in all CM subsets. A subset of CM PRGs (HSA^+^ALCAM^+^PDGFrα^+^) was found at E 9.5 and persisted in the At of E 13.5 and E 17.5 hearts (not represented). HSA^+^ CMs dramatically decrease around birth, coinciding with an increase in quiescent and binucleated Cav3^+^ cells, first detected at E 17.5. The adult myocardium is composed of 2 main CMs subsets: mononucleated HSA^+^Cav3^+^ and mono- and bi- nucleated Cav3^+^ CMs. Upon MI, HSA^+^ CMs frequency increases, and a small percentage of these is proliferating. Throughout cardiac maturation, proliferative CMs (↻) were only detected within the HSA^+^ compartment. Detailed information can be found in the text. ALCAM, activated leukocyte cell adhesion molecule; At, atria; Cav3, Caveolin 3; CD45, cluster of differentiation 45; CM, cardiomyocyte; E, embryonic day; Gp38, glycoprotein 38; HSA, heat stable antigen; ICAM-1, intercellular adhesion molecule 1; MCAM, melanoma cell adhesion molecule; MI, myocardial infarction; PDGFrα, platelet derived growth factor receptor alpha; PECAM-1, platelet/endothelial cell adhesion molecule 1; PRG, progenitor; Sca-1, stem cells antigen 1; Ter119, lymphocyte antigen 76 clone TER-119; Thy1, thymus cell antigen 1; Tnnt, troponin T.

We identified HSA, so far never associated to heart development or maturation, as a transversal marker of immature CMs throughout life. Our analysis showed a continuum in CM maturation, which is an asynchronous and apparently stochastic process that starts during development and can be prospectively identified by the expression of distinct surface markers (Fig 7). Immature CMs can be identified by a unique phenotype, i.e., HSA^+^MCAM^+^ALCAM^+^Cav3^−^; they progressively lose ALCAM and MCAM expression to become HSA^+^ only. HSA^+^ CMs decrease in frequency between E 17.5 and P7 but are the only CM subset that actively proliferates up to P7; they have spontaneously contractile properties in culture and are consistently mononucleated. Isolated from E 15 and P1 hearts, HSA^+^ CM engrafted cardiac tissue transplanted in the ear pinna of adult mice with a better efficiency than that of Cav3^+^ CMs.

The first signs of CM binucleation are observed around E 17.5 of development in Cav3^+^ CMs, but never in immature CMs (Tnnt^+^Cav3^−^) that decrease in frequency, coinciding with an increase in noncycling and binucleated Cav3^+^ CMs that compose the myocardium in adulthood. These results are in agreement with alterations endured by CMs during the first week of life, which encompass a transition from hyperplasia to hypertrophy and terminal differentiation [9,10]. This is also consistent with the higher expression of transcripts specific for cardiac structural proteins found at the single-cell level of Cav3^+^ postnatal compared with HSA^+^ CMs. By contrast, *Col3a1* that characterizes a recently described population of ECM expressing CMs detected after E 14.5 [28] was found in the present study in Cav3^+^ cells that we likewise also first detect after E13.5.

Interestingly, a higher frequency of immature HSA^+^ CMs (>70%) maintain cell integrity, after enzymatic digestion, compared with Cav3^+^ CMs (<4%). The observation that different subsets of postnatal CMs have different degrees of cell viability after enzymatic digestion is an important piece of information that impacts experiments performed with CM cell suspensions.

A subset (<1%) of adult CMs displayed HSA and remained mononucleated, thus resembling embryonic CM, despite expressing Cav3 at the cell surface. Postnatal and adult HSA^+^ cells share the same morphological features of immature CMs (small, round shaped and mononucleated). However, adult HSA^+^ CMs did not acquire spontaneous contractility in culture, indicating that they are less resistant than perinatal immature CMs to dissociation and thus more similar to Cav3^+^ CMs. Our findings are in line with previous reports showing low rates of cell division (0.76% [21] and 0.3% to 1% [42] per year) in small, mononucleated, and diploid CMs in the adult heart.

Foreseeing its therapeutic relevance, we tested whether HSA^+^ immature CMs could respond to a pathological challenge. We observed an upsurge in the frequency of adult mononucleated HSA^+^ CMs 7 days after MI (from 0.6% to 1.8%). This relative increase in HSA^+^ CMs can be explained by proliferation, detected at very low frequency in our analysis, by increased resistance of HSA^+^ CMs to hypoxia, by re-expression of HSA or by any combination of the above. In the developing heart, low oxygen tension is found in the compact myocardial layer [43] precisely where immature HSA^+^ CMs were found in this study. An adult CM subset protected from oxidative stress in hypoxic niches and exhibiting low proliferative activity upon injury has been recently identified. Similar to HSA^+^ CMs, these cells were mononucleated, small sized, and represented around 1% of the adult myocardium [42]. Although we have not found differences in the hypoxia regulated transcripts at steady state, we cannot rule out that both cell types might respond differently to ischemia.

Although HSA^+^ immature CMs do not proliferate sufficiently to regenerate the myocardium, they might account for the low CM turnover rate previously described in the adult [21,22,44] and might be more amenable than binucleated CMs to respond to mitotic stimuli. Importantly, using the strategy herein described, CMs at different maturation stages can now be prospectively isolated as viable cells from the adult heart, enabling further mechanistic studies.

## Materials and methods

### Ethics statement

All animal manipulations at i3S were approved by the Animal Ethics Committee and Direcção-Geral de Veterinária–DGAV; at Pasteur Institute, animal manipulations were approved by the Ethics Committee and by the French Agriculture ministry according to the Ethic Chart and the European Parliament Directive 2010/63/EU at both institutes.

### Mice

C57BL/6 mice (Charles River) 6 to 8 weeks old or timed pregnant females were used. Timed pregnancies were generated after overnight mating. The following morning, females with vaginal plug were considered to be at E 0.5. Ub–GFP mice used for transplantation experiments were a kind gift from P. Bousso (Pasteur Institute) [45].

### Mouse model of MI

MI was experimentally induced by permanent ligation of the left coronary artery as previously described by Nascimento and colleagues [46], and samples were analyzed 7 days after injury.

### Transplantation of Ub–GFP or CFSE^+^ cells in embryonic cardiac implants (ear-pinna model)

As shown in S8A Fig, E 15.5 cardiac Vt from WT embryos were dissected and grafted in the ear pinna of recipient adult WT mice, under anesthesia, as previously described by Ardehali and colleagues [26]. Seven days later, hearts from Ub–GFP (E 15.5) or from WT (E 15.5 and P1) mice were dissociated, and HSA^+^ immature CMs, Cav3^+^ CMs, or PDGFrα^+^ FBs (stromal cells) were sorted and directly injected into visible beating implants (10,000 cells per implant). In all experiments using P1 CMs, cells were additionally incubated for 30 minutes at 37°C with Hoechst 33342 (0.2 μg/ml, Molecular Probes H3570) and sorted as positive for DNA content (H^+^). After isolation, WT cells (E 15.5 or P1) were labeled with CFSE (Molecular Probes, C1157) as previously described by Tario and colleagues [27]. One week later, the implants were collected, and tissue was processed for immunofluorescence as described below.

### Isolation of live cardiac cells

Embryonic hearts were collected under a stereomicroscope, and the 3 anatomic heart structures (At, GV-AVJ, and Vt) were microdissected. Heart tissue was minced into 1 mm^3^ fragments and incubated for 15 minutes at 37°C in the enzymatic solution: for E 13.5 and E 17.5 hearts, 0.2 mg/mL collagenase (Sigma-Aldrich) in Hank's Balanced Salt Solution with calcium and magnesium (HBSS^+/+^, Invitrogen); for E 9.5 hearts, 0.1 mg/mL collagenase in HBSS^+/+^; for postnatal hearts 20 mM 2,3-Butanedione monoxime (BDM; Sigma-Aldrich) was added in all isolation steps to DPBS without Ca^2+^ and Mg^2+^(DPBS^−/−^, Invitrogen); and for adult hearts, 0.2 mg/mL collagenase with 20 mM BDM (Sigma-Aldrich) and with 60 U/mL DNase I (Roche, Switzerland). At the end of each round of digestion, tissue fragments were resuspended using a P1000 pipette (approximately 20 times). The remaining tissue was allowed to sediment, and the supernatant was collected in a tube containing the same volume of 10% FCS (Life Technologies)-HBSS^−/−^ and kept on ice while the digestion protocol continued. Digestion was repeated until no macroscopic tissue was detected. After digestion, cell suspensions were centrifuged 10 minutes, 290*g* at 4°C, resuspended in 1% FCS HBSS^−/−^, and filtered with a 70 μm mesh strainer (Fisher Scientific).

### Isolation of fixed CMs

Fixed CMs were isolated as described by Mollova and colleagues [47] with some alterations. E 13.5, E 17.5, P7, adult, and injured (MI or sham-operated) hearts were collected, washed in PBS (to remove blood, Invitrogen), minced into 2 mm^3^ pieces, flash frozen in liquid nitrogen, and stored at −80°C. For cell isolation, tissue pieces were fixed in 4% paraformaldehyde (Electron Microscopy Sciences) at room temperature for 2 hours, washed in PBS for 5 minutes, and digested with 3 mg/ml collagenase type II (Worthington) in HBSS on a rotator at 37°C until no macroscopic tissue was detected. Enzyme activity was blocked with 10% FBS-HBSS (Life Technologies). Cell suspensions were filtered through a 100-μm cell strainer (Fisher Scientific).

### Flow cytometry, cell sorting, and imaging flow cytometry

Heart-cell suspensions were stained with (conjugated or nonconjugated) antibodies (20 minutes, 4°C in the dark) followed by incubation with conjugated streptavidin (10 minutes, 4°C in the dark). Whenever using a nonconjugated antibody, a sequential incubation with a secondary antibody was performed for 15 minutes at 4°C in the dark (see S1 Table for the antibodies list). PI (1μg/ml) was used to exclude dead cells. In designated experiments of postnatal hearts, cells were incubated with Hoechst 33342 (0.2 μg/ml, Molecular Probes H3570) in 2% FBS-HBSS for 30 minutes at 37°C. Intracellular proteins (Ki67 and troponin) detection was performed after surface staining, and fixation and permeabilization with the Foxp3/Transcription Factor Staining Buffer Set (eBioscience, USA). DAPI (1/10000, Molecular Probes) was used to stain DNA in fixed cells (5 minutes at 4°C). EdU staining was also performed after surface staining, fixation, and permeabilization with the Click-it EdU flow cytometry assay kit (Molecular Probes) following the manufacturer’s procedures. Flow cytometry data were acquired in a BD FACSCanto II (BD Bioscience), BD LSRFortessa (BD Bioscience), and Sony SP6800 analyzer (Sony, Japan) and analyzed with the FlowJo version 10.0.8 (BD Bioscience), Kaluza 1.5 (Beckman Coulter), or R version 3.2.4 software (R foundation).

Cells were sorted in a BD FACSAria III directly into 96-well plates loaded with RT-STA Reaction mix (according to the manufacturer’s procedures, CellsDirect One-Step qRT-PCR Kit, Invitrogen) and 0.2× specific TaqMan Assay mix (see S3 Table for the TaqMan assays list, Thermofisher). For single-cell sorting, a control well with 20 cells was always used. Index-sorting tool on BD FACSDiva version 8.0.1 software (BD Bioscience) was activated to track and record the fluorescence data for each parameter of each individual cell collected in a precise position of the 96-well plate. This tool allowed the postsorting correlation of the levels of surface protein expression and the transcriptional profile (S2C Fig). Data were analyzed with the FlowJo version 10.0.8 software (BD Bioscience).

Fixed CMs were resuspended in BD Cytofix/Cytoperm Fixation/Permeabilization Kit (BD Biosciences), permeabilized in BD Perm/Wash buffer for 15 minutes, incubated with primary antibodies for 2 hours at 4°C, and incubated with Alexa Fluor-conjugated secondary antibodies for 30 minutes at 4°C. Prior to acquisition on ImageStream, nuclei were stained with 20 μM DRAQ5 (Biostatus, UK) and filtered with 100-μm cell strainer (Fisher Scientific). Data acquisition was performed using an Amnis ImageStreamX cytometer (Luminex). Files were collected with a cell classifier applied to the bright-field (BF) channel to capture events larger than 20 μm and included BF, FITC, PECy7, and DRAQ5 images. At least 30,000 cells were analyzed for each sample, and all images were captured with the 40× objective. Data analysis was performed with IDEAS software (version 6.0, Luminex). For each sample, only intact CMs, selected based on Actinin and DRAQ5 signal intensity, were considered for subsequent analysis. For the morphometric analysis, we applied a morphology mask to the BF channel, and for assessment of the number of nuclei per cell, we used the DRAQ5 images.

### Culture and live-cell imaging

E 15.5, P2, P4, and adult cardiac cells were isolated as above, and HSA^+^ cells were sorted following the gating strategy in S1A Fig. For the neonatal and adult cells, 20 mM BDM (Sigma-Aldrich) was added throughout the isolation procedure [48]. HSA^+^ cells were plated in 0.1% collagen (Life Technologies) for E 15.5 or in fibronectin/gelatin coated ibidi plates for postnatal cells, and cultured for 1 week in high-glucose Iscove's Modified Dulbecco's media (Life Technologies) supplemented with 20% FBS, 1× penicillin/streptomycin (Life Technologies, USA), 1× L-glutamine (Life Technologies), 50 μg/mL ascorbic acid, and 1.5 × 10^−4^ M 1-thioglycerol (Sigma-Aldrich), as previously described by Wu and colleagues [4]. Adult cardiac cells were incubated at 37°C in 3% O_2_. Live-cell imaging was performed on a temperature-controlled Zeiss Axiovert 200M microscope equipped with a CoolSnap HQ (Roper) camera (Zeiss, Germany). Sample position was controlled by an X-Y motorized stage, and images were acquired every 15 minutes using an A-Plan 20×/0.30 objective for 48 hours.

### Histological processing and immunofluorescence staining

Embryonic and adult (MI and sham-operated) hearts were fixed in 0.2% paraformaldehyde (Merck) overnight at 4°C, dehydrated in a sucrose gradient (4% followed of 15%), embedded in gelatin, and frozen. Tissue cryo-sections (4 μm thick) were blocked with either 4% FBS-1% BSA blocking solution or Vector M.O.M. basic kit (Vector Laboratories), depending on the specific conditions detailed in S2 Table. Tissue sections were incubated with primary antibodies overnight at 4°C, followed by 1-hour incubation with Alexa Fluor-conjugated secondary antibodies (see S2 Table for the antibodies list; Invitrogen). Slides were mounted, and nuclei were counterstained with aqueous mounting medium with DAPI (Vector Laboratories). Representative high-resolution images were acquired for each heart structure (At, GV-AVJ, and Vt) at 40× magnification in a confocal microscope (Leica SP5II, Leica, Germany). Whole-heart acquisitions were obtained using the high-content imaging system (IN Cell Analyzer 2000, GE Healthcare).

Isolated fixed CMs were resuspended in 10% FBS-PBS and spun onto superfrost slides in a cytocentrifuge (ThermoFisher). Cytospins were incubated with primary antibodies overnight at 4°C, followed by 1-hour incubation with Alexa Fluor-conjugated secondary antibodies. Acquired images were edited and quantified using the Image J version 1.51d software (NIH).

### Gene expression analysis

Sorted cells in RT-STA Reaction mix from the CellsDirectTM One-Step qRT-PCR Kit (Life Technologies) were kept at −80°C at least overnight before reverse transcription and specific target pre-amplification (20 cycles for single cells and 18 cycles for 20 cells). Pre-amplified samples were subjected to qRT-PCR (see S3 Table for Taqman assays list, Applied Biosystems) as previously described by Chea and colleagues [49].

### Bioinformatic analysis

Flow cytometry data analysis was performed in FCS files of live CD45^−^Ter119^−^CD31^−^ cell fraction using R package flowCore from R version 3.2.4 revised (2016-03-16 r70336) and the interface R Studio version 0.99.467 (R foundation) [50]. Subsequently, gating, as described in Fig 1 and in S1 Fig, was used to define each population. Map clustering of the flow cytometry data was performed using custom R scripts from R package t-SNE to dimensionality reduction − t-SNE (R foundation) [51] and Bioconductor.org package flowSOM to visualize Self-Organizing and Minimal Spanning Trees (Spanning Trees, R foundation) [52,53].

Gene expression raw data (BioMark Fluidigm, Applied Biosystems) of sorted cells at the population level was normalized with HPRT, and data are presented in 2^−ΔCt^. Single-cell gene expression analysis was performed in cells that expressed at least 1 of 3 housekeeping genes (*Hprt*, *Gapdh*, or *Actb*), and Ct values were used to the following analysis. A Ct value of 21 was the maximum value considered as expressed gene, and the background (i.e., nondetected) Ct value was 38. qRT-PCR data were processed with the QLUCORE (Qlucore AB 2008–2015, Sweden) software, and pheatmap package (version 1.0.10) R (R version 3.5.0, R foundation) was displayed in uncentered Pearson’s correlation unsupervised hierarchical clustering and PCA either for surface phenotype or transcripts.

### Statistical analysis

All results are shown as mean ± SD. Statistical significance was determined using the Student *t* test, except when comparing the frequency of HSA^+^ CMs from E 13.5 to P7 (one-way ANOVA followed by Tukey test). The statistical analysis of the data was performed using SigmaPlot software (*p* < 0.05 was considered statistically significant, R foundation) or QLUCORE (Qlucore AB 2008–2015, Sweden) software for the multidimensional analysis of multiplex qRT-PCR (two-way ANOVA, *p* = 0.007, *q* = 0.01).

## Supporting information

S1 Fig Gating strategy defining the cardiac cell populations. (A) Single-cell suspension from the 3 heart regions (At, GV-AVJ, and Vt) were analyzed for CD45, Ter119, PECAM-1, ICAM-1, Sca-1, Thy1, HSA, PDGFrα, ALCAM, and MCAM markers in a SP6800 Spectral analyzer. Representative contour plots of the indicated surface proteins in the CD45^−^Ter119^−^ fractions (the upper plots) and in the subsequent gates indicated by the black arrows are shown and define the gating strategy. Numbers indicate frequencies within the gates. (B) Listing of the complete surface signature for each cardiac population together with their acronyms and the corresponding color code. ALCAM, activated leukocyte cell adhesion molecule; At, atria; CD45, cluster of differentiation 45; GV-AVJ, great vessels and atrioventricular junction; HSA, heat stable antigen; ICAM-1, intercellular adhesion molecule 1; MCAM, melanoma cell adhesion molecule; PDGFrα, platelet derived growth factor receptor alpha; PECAM-1, platelet/endothelial cell adhesion molecule 1; Sca-1, stem cells antigen 1; Ter119, lymphocyte antigen 76 clone TER-119; Thy1, thymus cell antigen 1; Vt, ventricles.
(TIF)

S2 Fig Single-cell transcriptional profiles of cardiac populations. (A) Heat map displays the unsupervised hierarchical clustering analysis of the multiplex single-cell qRT-PCR data of individual cardiac cells (311 analyzed single cells) as in Fig 2. (B) PCA graph corresponding to the heat map analysis shown in (A). (C) Index-sorting analysis correlates the phenotype of each sorted cell with its transcriptional profile. Macroscopic view of the E 17.5 GV-AVJ dissected region showing the recurrent contamination with Vt tissue. Thy1 versus HSA dot plots showing the levels of Thy1 and HSA expression of each sorted cell, to which a number was ascribed. Heat map of the unsupervised hierarchical clustering for the multiplex single-cell qRT-PCR performed on the individually sorted cells. Using the index-sorting tool, we distinguished by the levels Thy1 expression Vt-derived CMs (low) from GV-AVJ HSA^+^ FBs (high). The underlying data in (A–D) can be found within S5 Data. CM, cardiomyocyte; E, embryonic day; FB, fibroblast; GV-AVJ, great vessels and atrioventricular junction; HSA, heat stable antigen; PCA, principal component analysis; qRT-PCR, quantitative real time polymerase chain reaction; Thy1, thymus cell antigen 1; Vt, ventricle.
(TIF)

S3 Fig Surface phenotype and cell cycle progression of the HSA+ CMs during heart morphogenesis. (A) Macroscopic view of embryonic hearts at E 9.5, E 13.5, and E 17.5 along with the respective dot plots of flow cytometry data from each heart region (At or PAt and Vt or PVt). Scale bar: 1 mm. (B) Cell cycle analysis of the main cardiac populations combining the surface markers herein identified. Intracellular Ki67 and DAPI allowed determining the frequency of cells in G_1_ (Ki67^+/−^ and DAPI^2N^; top/bottom left quadrants, red), in S/G_2_-M (Ki67^+^ and DAPI^2N>4N^, top right quadrant, blue), and in G_0_ (Ki67^−^ and DAPI^2N^, bottom left quadrant, green). Contour plots display E 9.5 whole-heart cells and E 13.5 and E 17.5 Vt cells. (C) Cell cycle analysis. G_1_ (Ki67^+/−^ and DAPI^2N^), S/G_2_-M (Ki67^+^ and DAPI^2N>4N^), G_0_ (Ki67^−^ and DAPI^2N^) and binucleated cells (Ki67^−^ and DAPI^4N^) of stromal (black gate), HSA^+^ CMs (salmon gate), and Cav3^+^ CMs (red gate) cardiac cells. (D) HSA and Cav3 expression in E 13.5, E 17.5, and P7 cardiac cells. Flow cytometry (left panels, *n* = 2) and cytospin (right panels, *n* = 3, 300 cells analyzed in each). (E) Cell cycle analysis as in (C) of P1, P5, and P15 HSA^+^ (upper panels) and Cav3^+^ (lower panels) CMs compared with P5 spleen cells. Scale bar: 20 μm. At, atria; Cav3, Caveolin-3; CM, cardiomyocyte; E, embryonic day; HSA, heat stable antigen; Ki67, Kiel clone 67; P, postnatal day; PAt, primitive atria; PVt, primitive ventricle; S/G_2_-M, synthesis phase/gap 2 phase-mitosis; Vt, ventricle.
(TIF)

S4 Fig Analysis of the 2 subsets of CMs for binucleation and Tnnt expression. (A) Representative contour plots of the height versus width in the Forward and Side Scatters, excluding the possibility of the 4N subset (binucleated Cav3^+^) to be result of cell doublets. (B) Demonstration of the Tnnt expression in both HSA^+^ and Cav3^+^ CM subsets. Because of a technical incompatibility to combine in the same staining Cav3 and Tnnt, we confirmed the presence of Tnnt in the 2 CM populations (HSA^+^ and Cav3^+^) after sorting. (C) Histograms of HSA and Cav3 expression in E 13.5, E 17.5, and P7 cardiac cells (flow cytometry; left panel, *n* = 2), of the frequency of cells exhibiting 1, 2, or more nuclei (cytology; right panels, *n* = 3; 300 cells analyzed in each) in Cav3^+^ cells (middle panel) and in HSA^+^ cells (right panel). The numbers of HSA^+^ cells analyzed were a mean of 10,000 in E 13.5, 5,000 in E 17.5, and 10 in P7, in each of 3 independent experiments. (D) qRT-PCR of P1 Cav3^+^H^+^ and Cav3^+^H^−^ cells. Ct value for the detection of HPRT per 100 cells used in each reaction (*n* = 3; 27 for Cav3^+^H^+^ cells and 32 for Cav3^+^H^−^, left graph). Tnnt2 expression after normalization for HPRT (right panel). The underlying data in (C−D) can be found within S6 Data. Cav3, Caveolin-3; CM, cardiomyocyte; Ct, cycle threshold value; E, embryonic day; HPRT, Hypoxanthine guanine phosphoribosyl transferase; HSA, heat stable antigen; qRT-PCR, quantitative real time polymerase chain reaction; Tnnt, troponin T.
(TIF)

S5 Fig HSA is expressed in all CMs and cardiac PRGs at E 9.5 onwards. (A) Coronal view of E 9.5 heart section stained for Actinin (red) and nuclear content (DAPI; blue), showing the 3 heart regions. Scale bar: 50 μm. Representative images of E 9.5 cardiac tissue display HSA coexpression with either Actinin (CMs, white *) or PECAM-1 (EndoCs, white #) in the primitive chambers and EndoC cushions, respectively. Scale bar: 20 μm. (B) Coronal view of E 13.5 heart section stained for Actinin (red) and nuclear content (DAPI, blue), showing the 3 heart regions. Scale bar: 50 μm. Representative images showing CMs (HSA^+^ Actinin^+^, insets). Scale bars: 20 μm for representative sections and 10 μm for insets. (C) Dissection of E 9.5 heart tube in the 3 main compartments (OFT, PVt, PAt). Flow cytometry plots with the expression of HSA in CD45, Ter119, PECAM-1, ICAM-1, and Sca-1 negative cell fraction of the OFT (left panel), PVt (middle panel), and PAt (right panel). CD45, cluster of differentiation 45; CM, cardiomyocyte; E, embryonic day; EndoC, endocardial cell; HSA, heat stable antigen; ICAM-1, intercellular adhesion molecule 1; OFT, outflow tract; PAt, primitive atria; PECAM-1, platelet/endothelial cell adhesion molecule 1; PRG, progenitor; PVt, primitive ventricle; Sca-1, stem cells antigen 1; Ter119, lymphocyte antigen 76 clone TER-119.
(TIF)

S6 Fig Purity analysis and quantification of the HSA^+^ CM cultures. (A) Representative contour plots with the gating strategy to isolate E 15.5 HSA^+^Cav3^−^ CMs (left plots) and control purity after sorting (right plots). Representative image of a CM in culture (upper right panel, see also MS1 and MS2). Scale: 20 μm. Frequency of contractile CMs in cultures (lower right panel). (B) Representative plots as in (A) for neonatal and adult cardiac cells (dot plots). Frequency of sorted cells that adhered to gelatin-coated plates (right panels), *n* = 4. Virtually all adherent cells were contractile and expressed cardiac troponin. No adherent cells were observed in cultures of Cav3^+^ CMs (more than 10,000 cells). The underlying data in (A−B) can be found within S7 Data. Cav3, Caveolin-3; CM, cardiomyocyte; E, embryonic day; HSA, heat stable antigen.
(TIF)

S7 Fig Immature CMs in the adult heart are mononucleated. (A) Image flow cytometry of HSA^−^ and HSA^+^ adult CMs. Binucleated CMs in the top panels show low levels of green that correspond to autofluorescence in the green channel. (B) Representative image of adult HSA^+^ CMs isolated from Ub–GFP mice after 48 hours in culture in 3% O_2_, stained for cardiac troponin (red), GFP (green), and DAPI. CM, cardiomyocyte; GFP, green fluorescent protein; HSA, heat stable antigen; Ub–GFP, Ubiquitin–GFP.
(TIF)

S8 Fig Ear-pinna transplantation experiments. (A) Schematic representation of the experimental design followed in the ear-pinna experiments. (B) Sorting strategy for the isolation of HSA^+^H^+^ and Cav3^+^H^+^ for transplantation (right panels). Purity of the transplanted populations (left panels). (C) Immunohistochemistry analysis for the expression of troponin (red), Ki67 (white), DAPI (blue), and GFP (Ub–GFP cells) of embryonic cardiac tissue implants injected with cardiac stromal cells from the E 15.5 Ub–GFP mice (upper panels, first strategy) and implants not injected as controls for the experiment described in Fig 4G (lower panels). Higher magnification of the region delimitated by the white rectangle (right panels). (D) Immunohistochemistry analysis for the expression of troponin (red), Ki67 (white), DAPI (blue), and CFSE (green) of embryonic cardiac tissue implants injected with E 15.5 or P1 WT HSA^+^H^+^CFSE^+^ cells (second strategy). Higher magnification of the region delimitated by the white rectangle (right panels). Scale bar: 50 μm. Cav3, Caveolin-3; CFSE, carboxyfluorescein succinimidyl ester; E, embryonic day; GFP, green fluorescent protein; HSA, heat stable antigen; Ki67, Kiel clone 67; Ub–GFP, Ubiquitin–GFP; WT, wild type.
(TIF)

S9 Fig Adult cardiac populations. (A) Macroscopic view of adult heart, depicting the dissected cardiac regions: At, GV-AVJ, and Vt. Scale bar: 1 mm. Radar plots of flow cytometry analysis in the CD45^−^ and Ter119^−^ cell fraction for the surface expression of HSA, Thy1, PECAM-1, ICAM-1, MCAM, ALCAM, and PDGFrα in the indicated heart regions (*n* = 2). (B) Flow cytometry profiles of adult whole-heart suspensions (the great vessels were dissected out) stained with Cav3, HSA, and Hoechst 33342, after exclusion of CD45-, Ter119-, PECAM-1−, PDGFrα-, and ICAM-1−expressing cells. Gating strategy and Hoechst 33342 (H^+^) expression in stromal cells (ICAM-1^+^, upper panel), HSA^+^ CMs (middle panel), and Cav3^+^ CMs (lower panel). (C) Heat map displays the unsupervised hierarchical clustering analysis of the multiplex qRT-PCR data in 100 sorted cells of the indicated adult cardiac populations (*n* = 3). (D) Representative adult heart sections of the 3 heart regions (At, GV-AVJ, and Vt) stained for Actinin (red) and nuclear content (DAPI; blue), showing (1) FBs (PDGFrα^+^ cells; top row), (2) EpiCs (Gp38^+^ cells), and (3) EPDCs (ICAM-1^+^ cells); bottom rows. Scale bar: 20 μm. (E) Representative adult heart sections 7 days post MI stained for Actinin (red) and nuclear content (DAPI; blue), showing (a) the cellular infiltrate in the peri-infarcted region (CD45^+^ hematopoietic cells and the extracellular matrix protein TNC); and coexpression of HSA with (b) hematopoietic cells (CD45^+^), (c) ECs (PECAM-1^+^), and (d) SMCs (SMA^+^PDGFrα^+^); scale bar: 20 μm. The underlying data in (B) can be found within S8 Data. ALCAM, activated leukocyte cell adhesion molecule; At, atria; Cav3, Caveolin-3; CD45, cluster of differentiation 45; CM, cardiomyocyte; EC, endothelial cell; EPDC, epicardial-derived cell; EpiC, epicardial cell; FB, fibroblast; Gp38, glycoprotein 38; GV-AVJ, great vessels and atrioventricular junction; HSA, heat stable antigen; ICAM-1, intercellular adhesion molecule 1; MCAM, melanoma cell adhesion molecule; MI, myocardial infarction; PDGFrα, platelet derived growth factor receptor alpha; PECAM-1, platelet/endothelial cell adhesion molecule 1; qRT-PCR, quantitative real time polymerase chain reaction; SMA, smooth muscle actin; SMC, smooth muscle cell; Ter119, lymphocyte antigen 76 clone TER-119; Thy1, thymus cell antigen 1; TNC, TenascinC; Vt, ventricle.
(TIF)

S1 Table List of antibodies used in flow cytometry. (DOCX)

S2 Table List of antibodies used in immunofluorescence. (DOCX)

S3 Table List of Taqman assays. (DOCX)

S1 Data Ct values from qRT-PCR amplification that generated the data shown in Fig 2A and 2B. Individual Ct values obtained in the Biomark platform for the amplification of 20 cells in each population for the designated transcript (rows A, B) from the designated E 17.5 heart populations (columns C-AO). Ct, cycle threshold value; E, embryonic day.
(XLSX)

S2 Data HSA^+^ CMs. The numbers of HSA^+^ CMs (± SD) counted in E 13.5, E 17.5, and P7 shown in Fig 4E in 3 independent experiments. Number of CFSE^+^ CMs (E15.5 and P1 HSA^+^ Hoechst^+^ or Cav3^+^ Hoechst^+^) counted per section, in 10 consecutive sections, in 2 independent experiments shown in Fig 4G. Cav3, Caveolin-3; CFSE,; CM, cardiomyocyte; E, embryonic day; HSA, heat stable antigen; P, postnatal day.
(XLSX)

S3 Data P1 CM single-cell multiplex qRT-PCR. Ct values obtained in single-cell multiplex qRT-PCR using the Biomark platform for the indicated genes (columns) for P1 Cav3^+^ or HSA^+^ CMs (rows) shown in Fig 5A. Frequencies of single CMs (P1 Cav3^+^ or HSA^+^) expressing the indicated transcripts (columns) shown in Fig 5B. Ct values of individual HSA^+^ or Cav3^+^ CMs shown in Fig 5C and corresponding to the amplification shown in Fig 5A. Cav3, Caveolin-3; CM, cardiomyocyte; Ct, cycle threshold value; HSA, heat stable antigen; P, postnatal day; qRT-PCR, quantitative real time polymerase chain reaction.
(XLSX)

S4 Data Adult CMs. Ct values of the single adult HSA+ CMs (columns) multiplex qRTPCR using the Biomark platform for the genes in rows 2–44 shown in Fig 6B. Average (± SDs) of HSA^+^ CMs counted in sections of MI or sham-operated mice and average (± SDs) of Ki67^+^ HSA^+^, and HSA^−^ CMs shown in Fig 6C. Mean area μm^2^ and mean length (μm, ±SDs) of adult HSA^−^ and HSA^+^ CMs shown in Fig 6D. Frequencies (± SDs) of HSA^+^ CMs counted in Image stream analysis of infarcted or sham-operated hearts in 3 independent experiments shown in Fig 6E. CM, cardiomyocyte; HSA, heat stable antigen; Ki67, Kiel clone 67; MI, myocardial infarction; qRT-PCR, quantitative real time polymerase chain reaction.
(XLSX)

S5 Data Single-cell qRT-PCR of cardiac cells at different stages of gestation. Ct values of single-cell qRT-PCR analysis for the genes in designated in rows using the Biomark platform of different cell populations (indicated in columns, 13, E 13.5; 9, E 9.5; 17, E 17.5; A; V; OFT; AVJ; the phenotype is mentioned followed by the number of the respective cell that can be traced to the flow cytometry plot through the index sorting) shown in S2A and S2B Fig. Ct values of single-cell qRT-PCR using the Biomark platform for the genes designated in rows, for 11 single cells referring to the index sorting in S2C Fig and corresponding to CMs or AVJ FBs. Ct values of single-cell qRT-PCR using the Biomark platform for the genes designated in rows, for HSA^+^ CMs isolated from E 9.5, E 13.5, and E 17.5 as shown in S2D Fig. A, atria; AVJ, atrioventricular junction; Ct, cycle threshold value; E, embryonic day; FB, fibroblast; HSA, heat stable antigen; OFT, outflow tract; qRT-PCR, quantitative real time polymerase chain reaction; V, ventricle.
(XLSX)

S6 Data Mono- and binucleated CMs. Frequency (± SD) of HSA^+^, Cav3^+^, and HSA^+^ Cav3^+^ CMs and frequency (± SD) of mononucleated, binucleated, and polynucleated CMs found in E 13.5, E 17.7, and P7 and shown in S4C Fig. Ct values found in cDNA from 100 cells either Cav3^+^ Hoechst^+^ or Cav3^+^ Hoechst^−^ from P1 hearts shown in S4D Fig. Cav3, Caveolin-3; CM, cardiomyocyte; Ct, cycle threshold value; E, embryonic day; HSA, heat stable antigen; P, postnatal day.
(XLSX)

S7 Data CMs in culture. Frequency of E 15.5 HSA^+^ CMs with contractile properties in culture shown in S6A Fig. Number of positive wells (response) for the growth of CMs in 6–20 wells per dilution (cell numbers per well-dose) and per population (in column A) shown in S6B Fig. CM, cardiomyocyte; E, embryonic day; HSA, heat stable antigen.
(XLSX)

S8 Data qRT-PCR of adult cardiac cells. Ct values of 100 adult cardiac cells per well for the indicated genes (rows) The phenotype of the cells is designated as ICAM-1^+^, PDGFrα^+^, or negative for both, DN shown in S9B Fig. A, atria; AVJ, atrioventricular junction (followed by the number of the cell); Ct, cycle threshold value; DN, double negative; ICAM-1, intercellular adhesion molecule 1; PDGFrα, platelet derived growth factor receptor alpha; qRT-PCR, quantitative real time polymerase chain reaction; V, ventricle.
(XLSX)

S1 Movie E 15.5 HSA^+^ CM dividing. Representative live-cell imaging of HSA^+^ CMs isolated from E 15.5 hearts dividing in culture. Time, hour:minute. Scale bar: 20 μm. CM, cardiomyocyte; E, embryonic day; HSA, heat stable antigen.
(AVI)

S2 Movie E 15.5 HSA^+^ CM beating. Representative example of a contractile HSA^+^ CM isolated from E 15.5 hearts. Time, minute:second. Scale bar: 20 μm. CM, cardiomyocyte; E, embryonic day; HSA, heat stable antigen.
(AVI)

S3 Movie P2 HSA^+^ CM beating. Representative example of a contractile HSA^+^ CM isolated from P2 hearts. Time, minute:second. Scale bar: 20 μm. CM, cardiomyocyte; HSA, heat stable antigen; P, postnatal day.
(AVI)

S4 Movie P4 HSA^+^ CM beating. Representative example of a contractile HSA^+^ CM isolated from P4 hearts. Time, minute:second. Scale bar: 20 μm. CM, cardiomyocyte; HSA, heat stable antigen; P, postnatal day.
(AVI)

## Abbreviations

ALCAM: activated leukocyte cell adhesion molecule
BDM: 2,3-Butanedione monoxime
BF: bright field
Cav3: Caveolin-3
CD24: cluster of differentiation 24
CD45: cluster of differentiation 45
CFSE: carboxyfluorescein succinimidyl ester
c-Kit: proto-oncogene receptor tyrosine kinase
CM: cardiomyocyte
Ct: cycle threshold value
Ddr2: discoidin domain receptor family member 2
DN: double negative
E: embryonic day
EC: endothelial cell
ECM: extracellular matrix
EdU: 5-ethynyl-2´-deoxyuridine
EMT: epithelial-to-mesenchymal transition
EndoC: endocardial cell
EPDC: epicardial-derived cell
EpiC: epicardial cell
FC: flow cytometry
FB: fibroblast
FHF: first heart field
G_1_: gap 1 phase
GFP: green fluorescent protein
Gp38: glycoprotein 38
GV-AVJ: great vessels and atrioventricular junction
HBSS: Hank's Balanced Salt Solution
HPRT: Hypoxanthine guanine phosphoribosyl transferase
HSA: heat stable antigen
Ki67: Kiel clone 67
MCAM: melanoma cell adhesion molecule
ICAM-1: intercellular adhesion molecule 1
MI: myocardial infarction
OFT: outflow tract
P: postnatal day
PAt: primitive atria
PCA: principal component analysis
PDGFrα: platelet derived growth factor receptor alpha
PDGFrβ: platelet derived growth factor receptor beta
PECAM-1: platelet/endothelial cell adhesion molecule 1
PI: propidium iodide
PRG: progenitor
qRT-PCR: quantitative real time polymerase chain reaction
PVt: primitive ventricle
Sca-1: stem cells antigen 1
S/G_2_-M: synthesis phase/gap 2 phase-mitosis
SHF: second heart field
SIRPα: signal regulatory protein alpha
SMA: smooth muscle actin
SMC: smooth muscle cell
Ter119: lymphocyte antigen 76 clone TER-119
Thy1: thymus cell antigen 1
Tnnt: troponin T
t-SNE: t-distributed stochastic neighbor embedding
Ub−GFP: ubiquitin−GFP
VCAM-1: vascular cell adhesion molecule 1
WT: wild type
