# Supplementary figures and images for: Mouse HSA+ immature cardiomyocytes persist in the adult heart and expand after ischemic injury
Source: PLoS Biol. 2019 Jun 27;17(6):e3000335. doi: 10.1371/journal.pbio.3000335 (PMC6619826; doi:10.1371/journal.pbio.3000335)

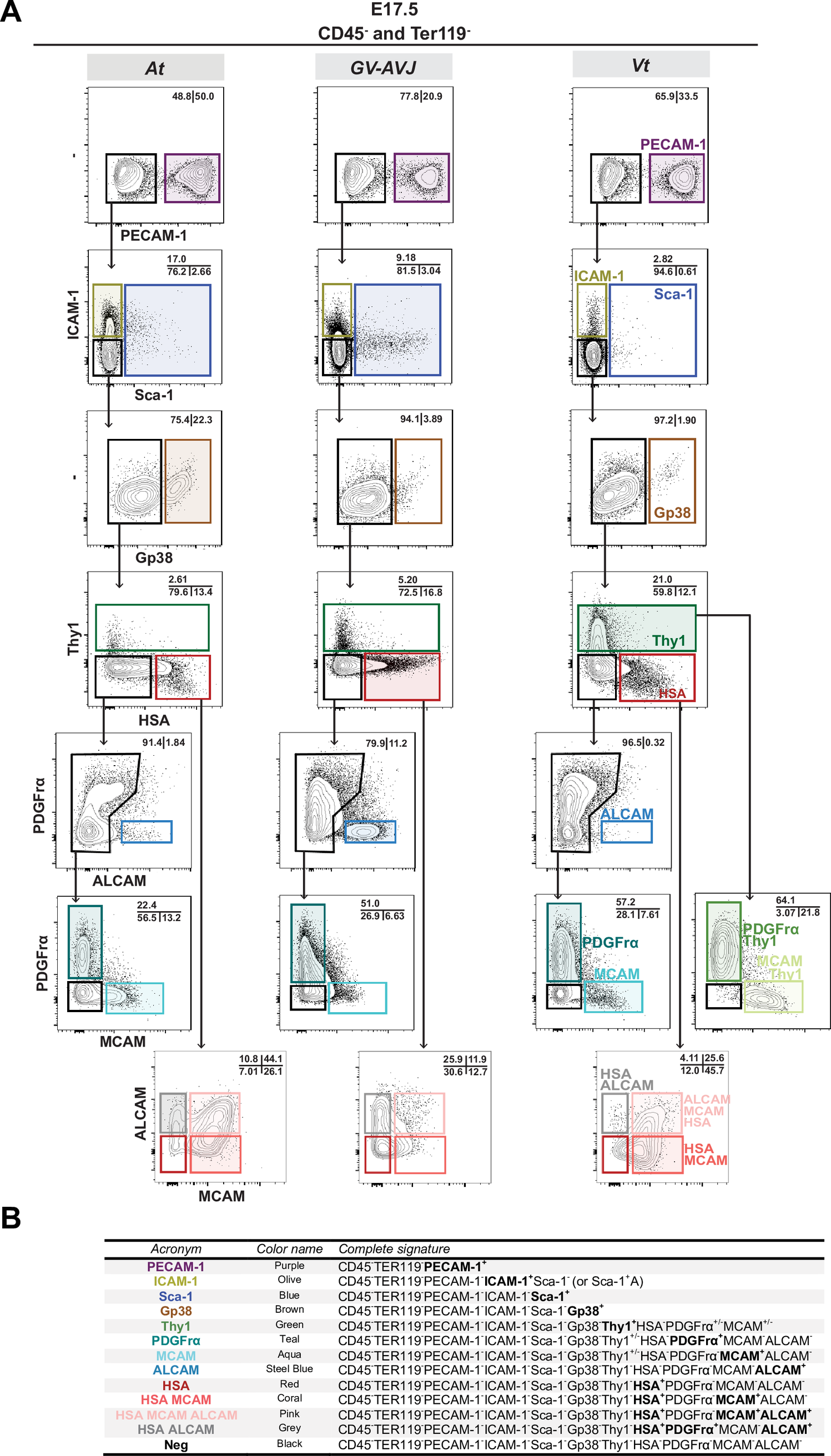

Supplement: S1 Fig — (A) Single-cell suspension from the 3 heart regions (At, GV-AVJ, and Vt) were analyzed for CD45, Ter119, PECAM-1, ICAM-1, Sca-1, Thy1, HSA, PDGFrα, ALCAM, and MCAM markers in a SP6800 Spectral analyzer. Representative contour plots of the indicated surface proteins in the CD45−Ter119− fractions (the upper plots) and in the subsequent gates indicated by the black arrows are shown and define the gating strategy. Numbers indicate frequencies within the gates. (B) Listing of the complete surface signature for each cardiac population together with their acronyms and the corresponding color code. ALCAM, activated leukocyte cell adhesion molecule; At, atria; CD45, cluster of differentiation 45; GV-AVJ, great vessels and atrioventricular junction; HSA, heat stable antigen; ICAM-1, intercellular adhesion molecule 1; MCAM, melanoma cell adhesion molecule; PDGFrα, platelet derived growth factor receptor alpha; PECAM-1, platelet/endothelial cell adhesion molecule 1; Sca-1, stem cells antigen 1; Ter119, lymphocyte antigen 76 clone TER-119; Thy1, thymus cell antigen 1; Vt, ventricles. (TIF) [file pbio.3000335.s001.tif]

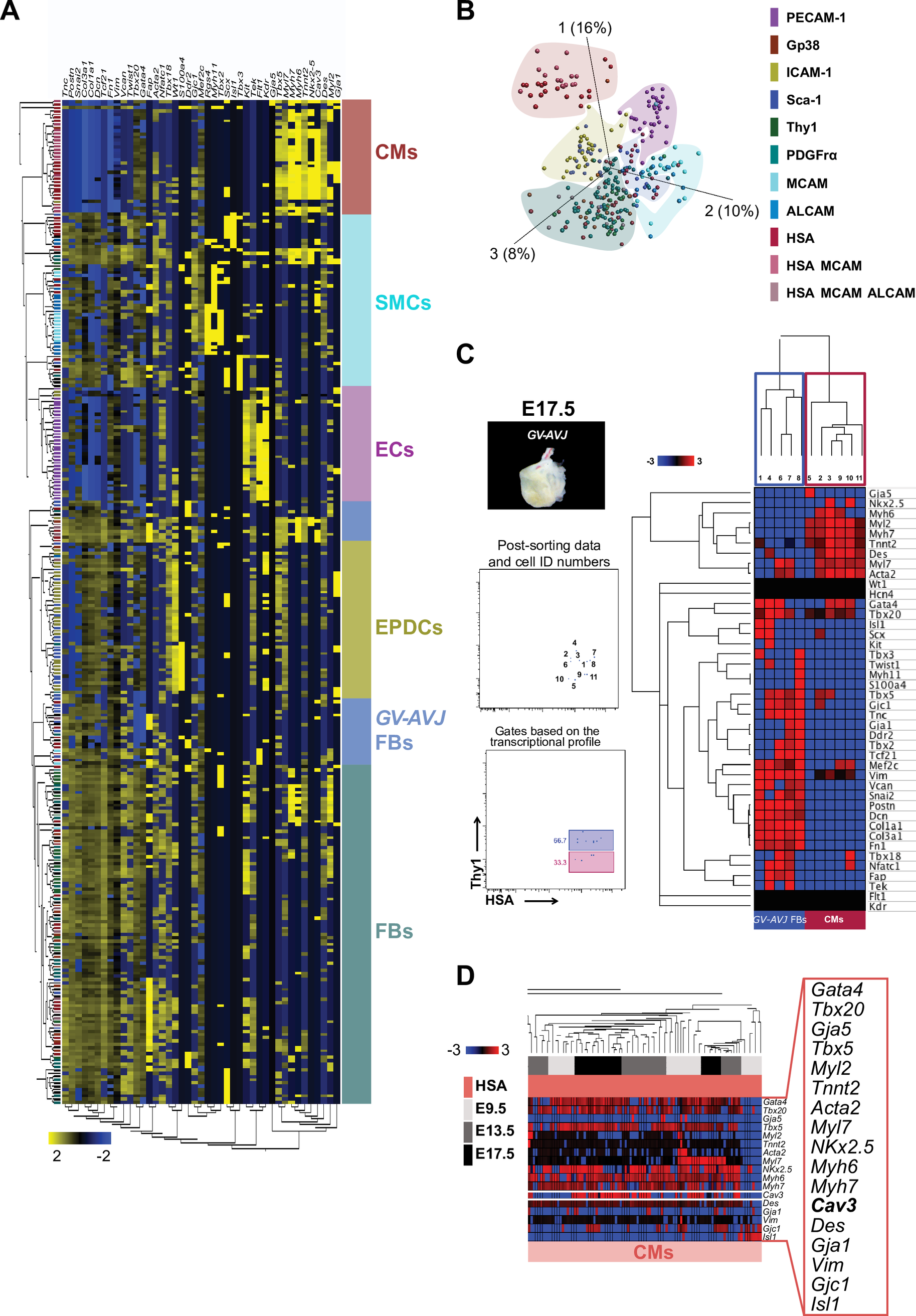

Supplement: S2 Fig — (A) Heat map displays the unsupervised hierarchical clustering analysis of the multiplex single-cell qRT-PCR data of individual cardiac cells (311 analyzed single cells) as in Fig 2. (B) PCA graph corresponding to the heat map analysis shown in (A). (C) Index-sorting analysis correlates the phenotype of each sorted cell with its transcriptional profile. Macroscopic view of the E 17.5 GV-AVJ dissected region showing the recurrent contamination with Vt tissue. Thy1 versus HSA dot plots showing the levels of Thy1 and HSA expression of each sorted cell, to which a number was ascribed. Heat map of the unsupervised hierarchical clustering for the multiplex single-cell qRT-PCR performed on the individually sorted cells. Using the index-sorting tool, we distinguished by the levels Thy1 expression Vt-derived CMs (low) from GV-AVJ HSA+ FBs (high). The underlying data in (A–D) can be found within S5 Data. CM, cardiomyocyte; E, embryonic day; FB, fibroblast; GV-AVJ, great vessels and atrioventricular junction; HSA, heat stable antigen; PCA, principal component analysis; qRT-PCR, quantitative real time polymerase chain reaction; Thy1, thymus cell antigen 1; Vt, ventricle. (TIF) [file pbio.3000335.s002.tif]

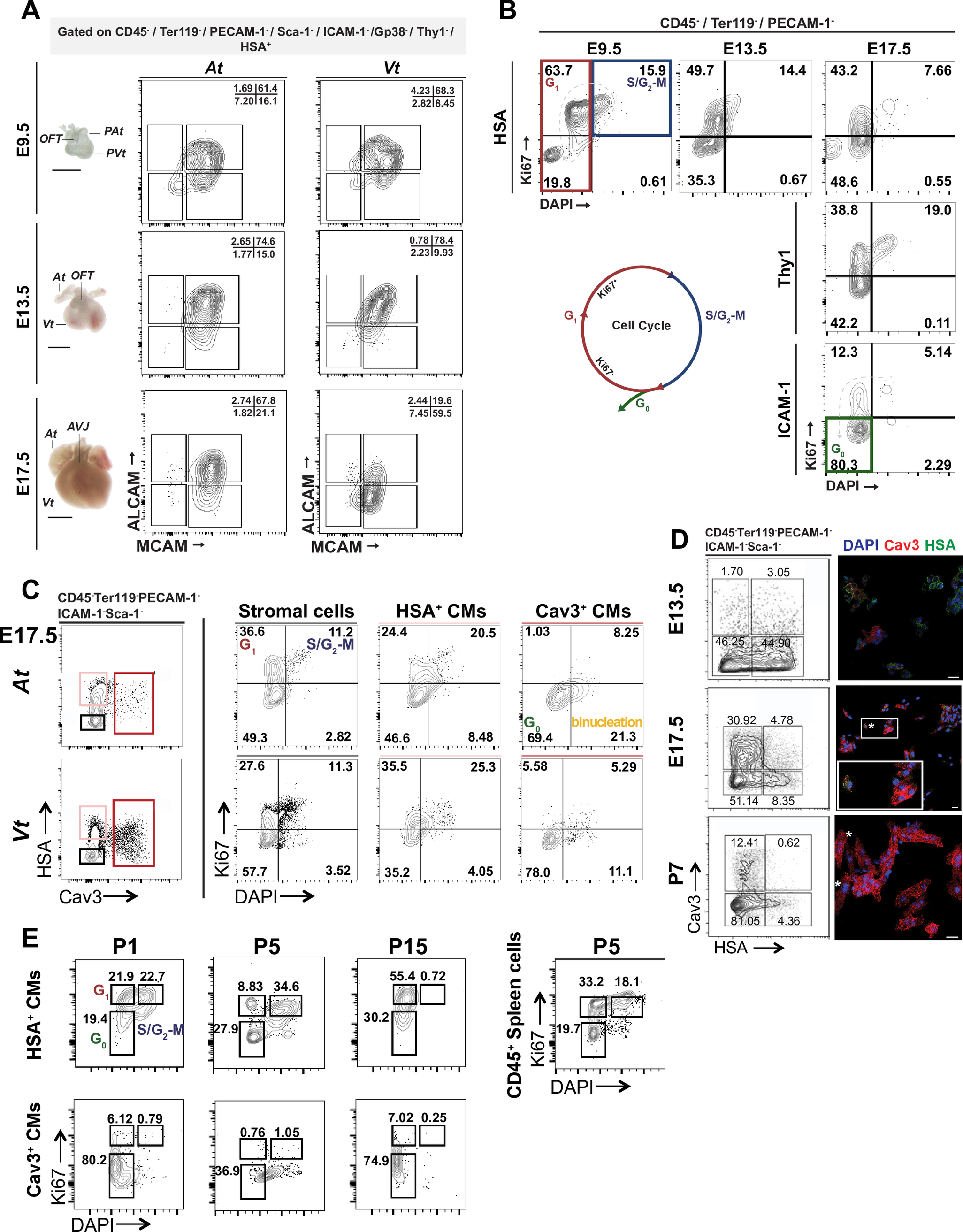

Supplement: S3 Fig — (A) Macroscopic view of embryonic hearts at E 9.5, E 13.5, and E 17.5 along with the respective dot plots of flow cytometry data from each heart region (At or PAt and Vt or PVt). Scale bar: 1 mm. (B) Cell cycle analysis of the main cardiac populations combining the surface markers herein identified. Intracellular Ki67 and DAPI allowed determining the frequency of cells in G1 (Ki67+/− and DAPI2N; top/bottom left quadrants, red), in S/G2-M (Ki67+ and DAPI2N>4N, top right quadrant, blue), and in G0 (Ki67− and DAPI2N, bottom left quadrant, green). Contour plots display E 9.5 whole-heart cells and E 13.5 and E 17.5 Vt cells. (C) Cell cycle analysis. G1 (Ki67+/− and DAPI2N), S/G2-M (Ki67+ and DAPI2N>4N), G0 (Ki67− and DAPI2N) and binucleated cells (Ki67− and DAPI4N) of stromal (black gate), HSA+ CMs (salmon gate), and Cav3+ CMs (red gate) cardiac cells. (D) HSA and Cav3 expression in E 13.5, E 17.5, and P7 cardiac cells. Flow cytometry (left panels, n = 2) and cytospin (right panels, n = 3, 300 cells analyzed in each). (E) Cell cycle analysis as in (C) of P1, P5, and P15 HSA+ (upper panels) and Cav3+ (lower panels) CMs compared with P5 spleen cells. Scale bar: 20 μm. At, atria; Cav3, Caveolin-3; CM, cardiomyocyte; E, embryonic day; HSA, heat stable antigen; Ki67, Kiel clone 67; P, postnatal day; PAt, primitive atria; PVt, primitive ventricle; S/G2-M, synthesis phase/gap 2 phase-mitosis; Vt, ventricle. (TIF) [file pbio.3000335.s003.tif]

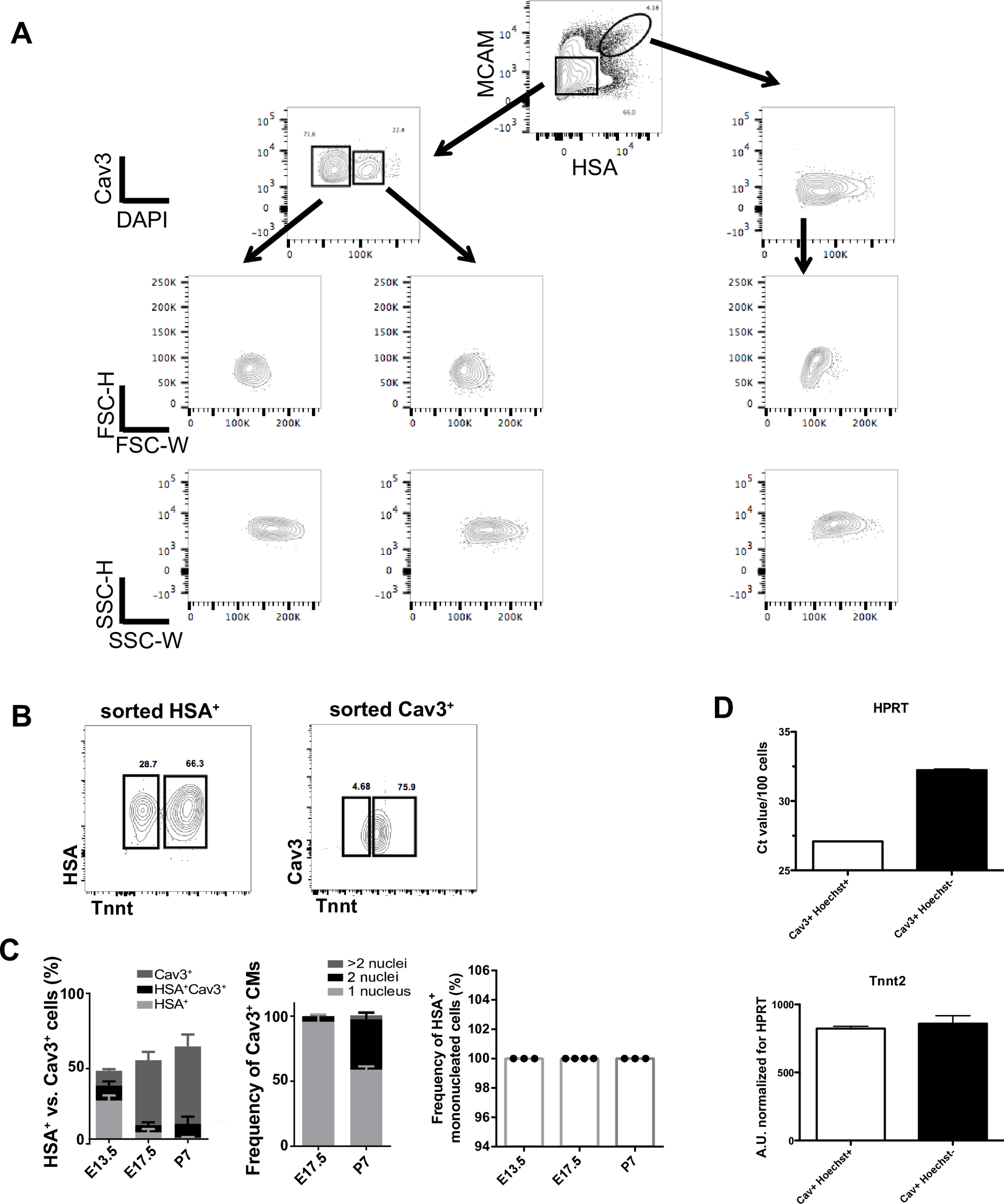

Supplement: S4 Fig — (A) Representative contour plots of the height versus width in the Forward and Side Scatters, excluding the possibility of the 4N subset (binucleated Cav3+) to be result of cell doublets. (B) Demonstration of the Tnnt expression in both HSA+ and Cav3+ CM subsets. Because of a technical incompatibility to combine in the same staining Cav3 and Tnnt, we confirmed the presence of Tnnt in the 2 CM populations (HSA+ and Cav3+) after sorting. (C) Histograms of HSA and Cav3 expression in E 13.5, E 17.5, and P7 cardiac cells (flow cytometry; left panel, n = 2), of the frequency of cells exhibiting 1, 2, or more nuclei (cytology; right panels, n = 3; 300 cells analyzed in each) in Cav3+ cells (middle panel) and in HSA+ cells (right panel). The numbers of HSA+ cells analyzed were a mean of 10,000 in E 13.5, 5,000 in E 17.5, and 10 in P7, in each of 3 independent experiments. (D) qRT-PCR of P1 Cav3+H+ and Cav3+H− cells. Ct value for the detection of HPRT per 100 cells used in each reaction (n = 3; 27 for Cav3+H+ cells and 32 for Cav3+H−, left graph). Tnnt2 expression after normalization for HPRT (right panel). The underlying data in (C−D) can be found within S6 Data. Cav3, Caveolin-3; CM, cardiomyocyte; Ct, cycle threshold value; E, embryonic day; HPRT, Hypoxanthine guanine phosphoribosyl transferase; HSA, heat stable antigen; qRT-PCR, quantitative real time polymerase chain reaction; Tnnt, troponin T. (TIF) [file pbio.3000335.s004.tif]

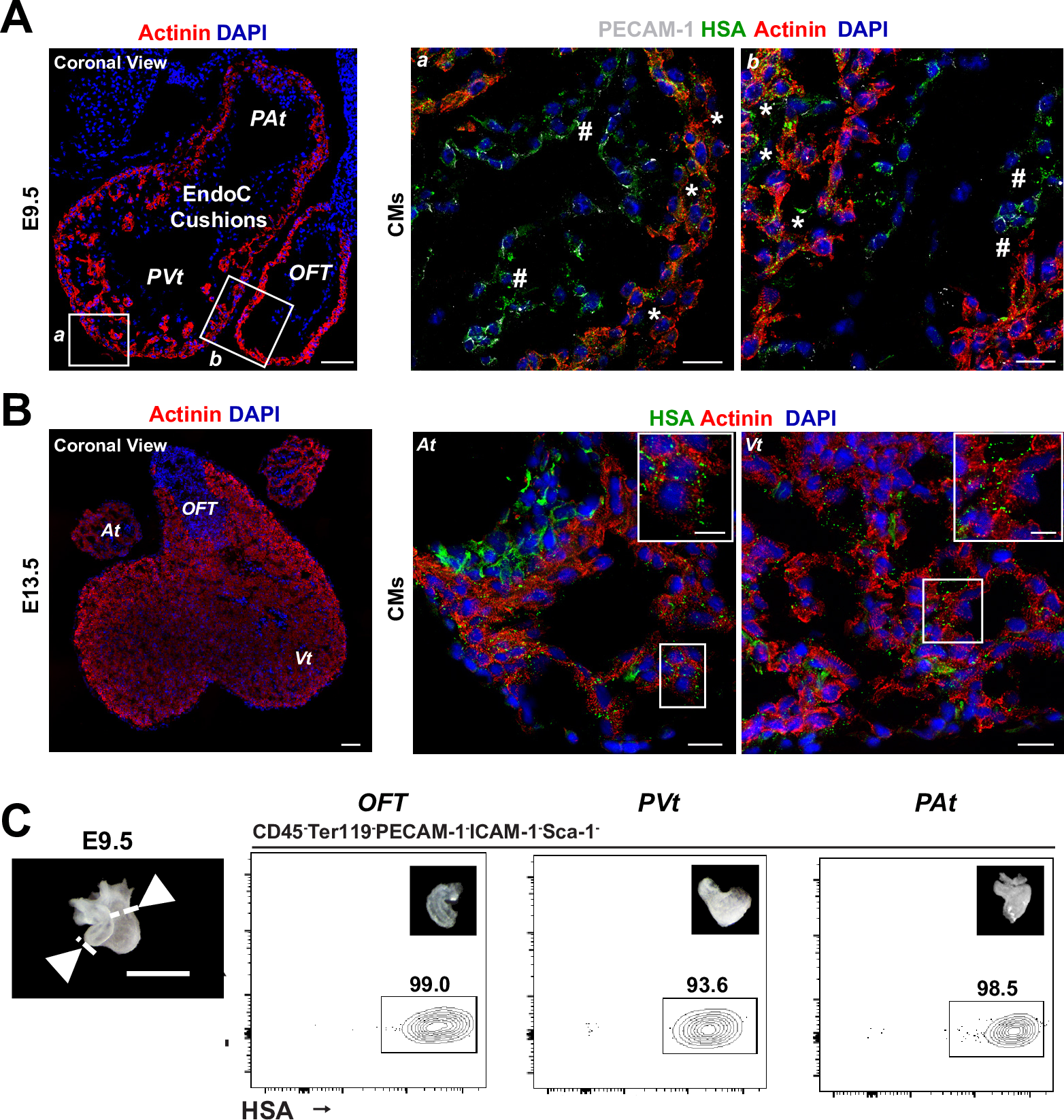

Supplement: S5 Fig — (A) Coronal view of E 9.5 heart section stained for Actinin (red) and nuclear content (DAPI; blue), showing the 3 heart regions. Scale bar: 50 μm. Representative images of E 9.5 cardiac tissue display HSA coexpression with either Actinin (CMs, white *) or PECAM-1 (EndoCs, white #) in the primitive chambers and EndoC cushions, respectively. Scale bar: 20 μm. (B) Coronal view of E 13.5 heart section stained for Actinin (red) and nuclear content (DAPI, blue), showing the 3 heart regions. Scale bar: 50 μm. Representative images showing CMs (HSA+ Actinin+, insets). Scale bars: 20 μm for representative sections and 10 μm for insets. (C) Dissection of E 9.5 heart tube in the 3 main compartments (OFT, PVt, PAt). Flow cytometry plots with the expression of HSA in CD45, Ter119, PECAM-1, ICAM-1, and Sca-1 negative cell fraction of the OFT (left panel), PVt (middle panel), and PAt (right panel). CD45, cluster of differentiation 45; CM, cardiomyocyte; E, embryonic day; EndoC, endocardial cell; HSA, heat stable antigen; ICAM-1, intercellular adhesion molecule 1; OFT, outflow tract; PAt, primitive atria; PECAM-1, platelet/endothelial cell adhesion molecule 1; PRG, progenitor; PVt, primitive ventricle; Sca-1, stem cells antigen 1; Ter119, lymphocyte antigen 76 clone TER-119. (TIF) [file pbio.3000335.s005.tif]

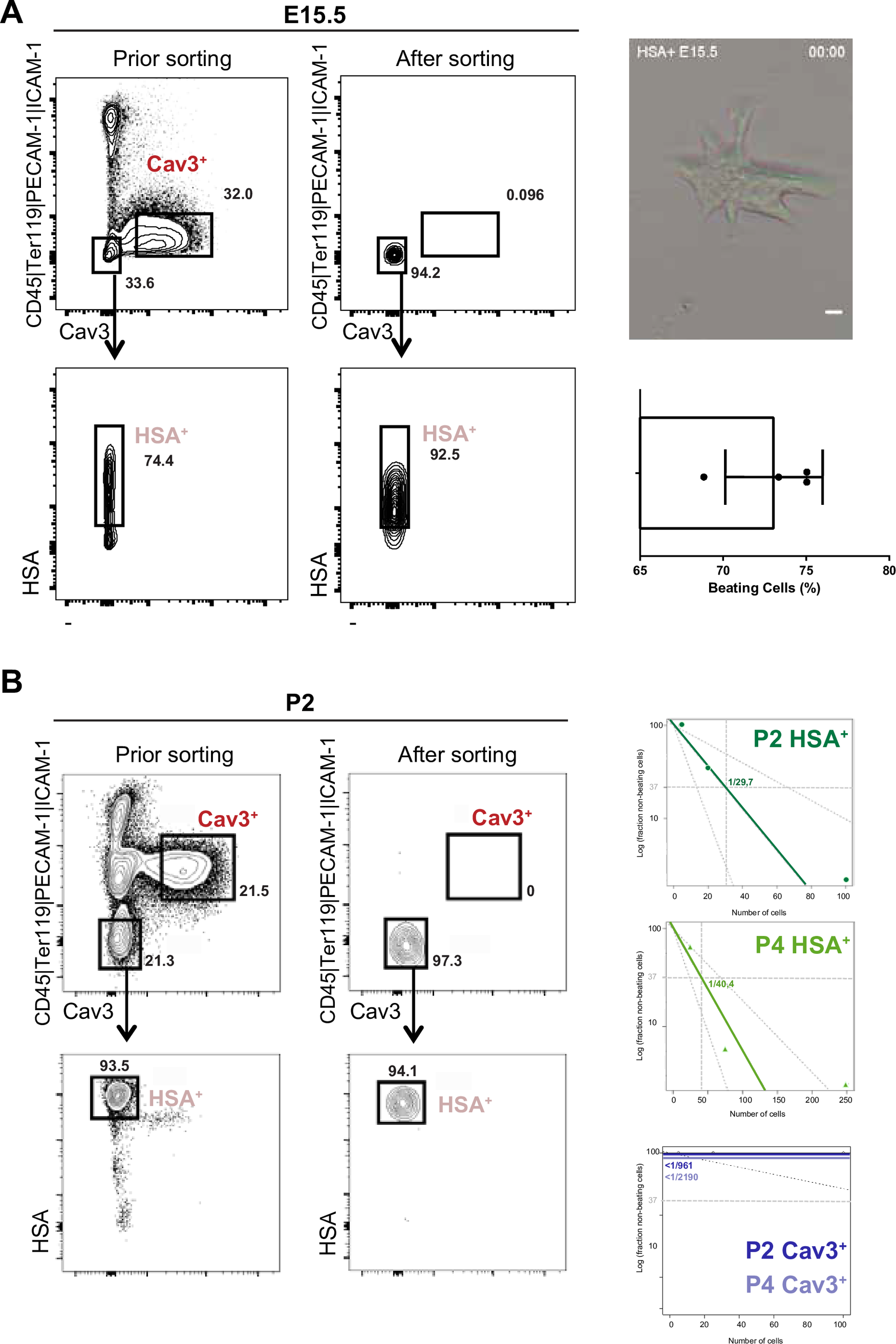

Supplement: S6 Fig — (A) Representative contour plots with the gating strategy to isolate E 15.5 HSA+Cav3− CMs (left plots) and control purity after sorting (right plots). Representative image of a CM in culture (upper right panel, see also MS1 and MS2). Scale: 20 μm. Frequency of contractile CMs in cultures (lower right panel). (B) Representative plots as in (A) for neonatal and adult cardiac cells (dot plots). Frequency of sorted cells that adhered to gelatin-coated plates (right panels), n = 4. Virtually all adherent cells were contractile and expressed cardiac troponin. No adherent cells were observed in cultures of Cav3+ CMs (more than 10,000 cells). The underlying data in (A−B) can be found within S7 Data. Cav3, Caveolin-3; CM, cardiomyocyte; E, embryonic day; HSA, heat stable antigen. (TIF) [file pbio.3000335.s006.tif]

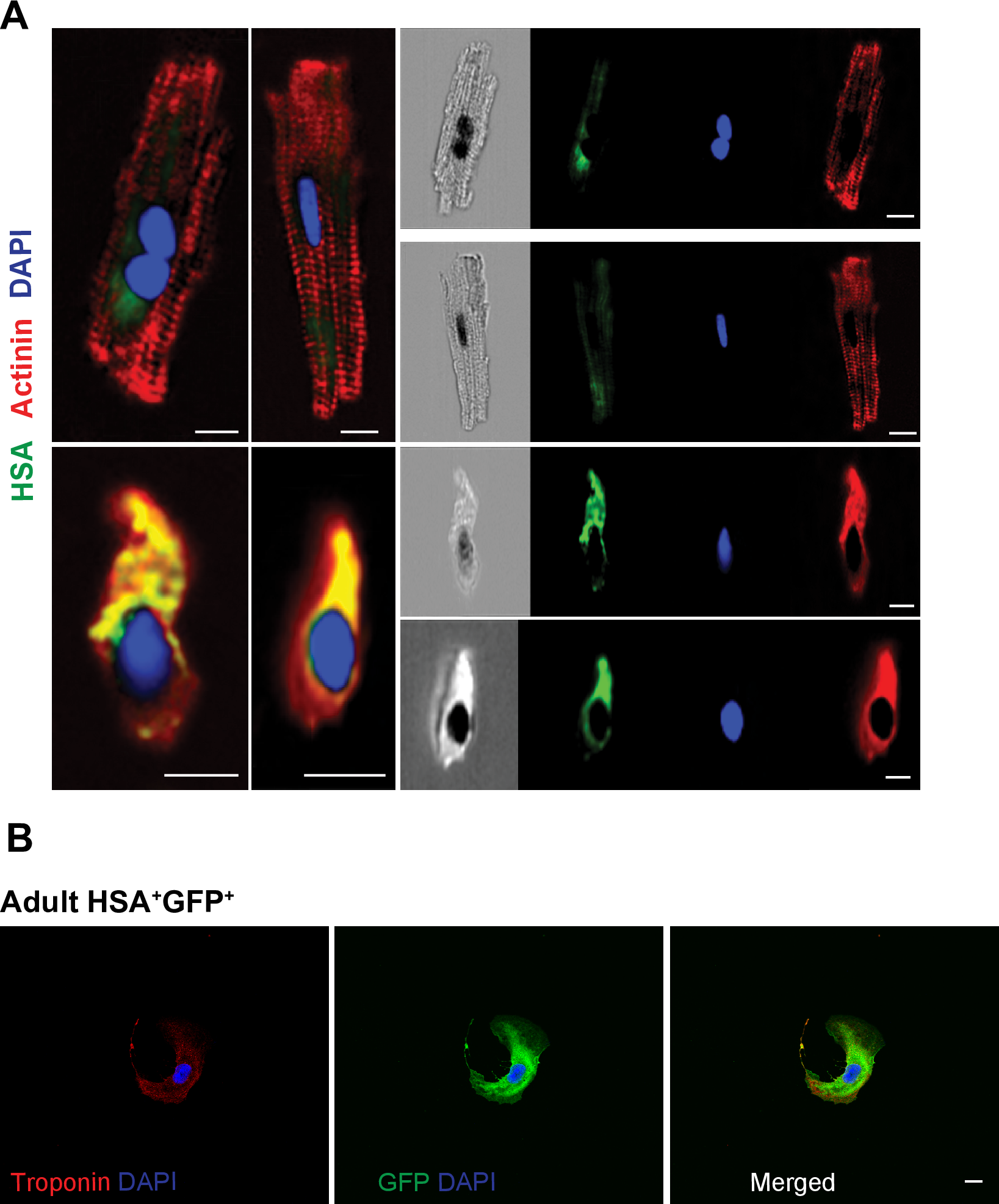

Supplement: S7 Fig — (A) Image flow cytometry of HSA− and HSA+ adult CMs. Binucleated CMs in the top panels show low levels of green that correspond to autofluorescence in the green channel. (B) Representative image of adult HSA+ CMs isolated from Ub–GFP mice after 48 hours in culture in 3% O2, stained for cardiac troponin (red), GFP (green), and DAPI. CM, cardiomyocyte; GFP, green fluorescent protein; HSA, heat stable antigen; Ub–GFP, Ubiquitin–GFP. (TIF) [file pbio.3000335.s007.tif]

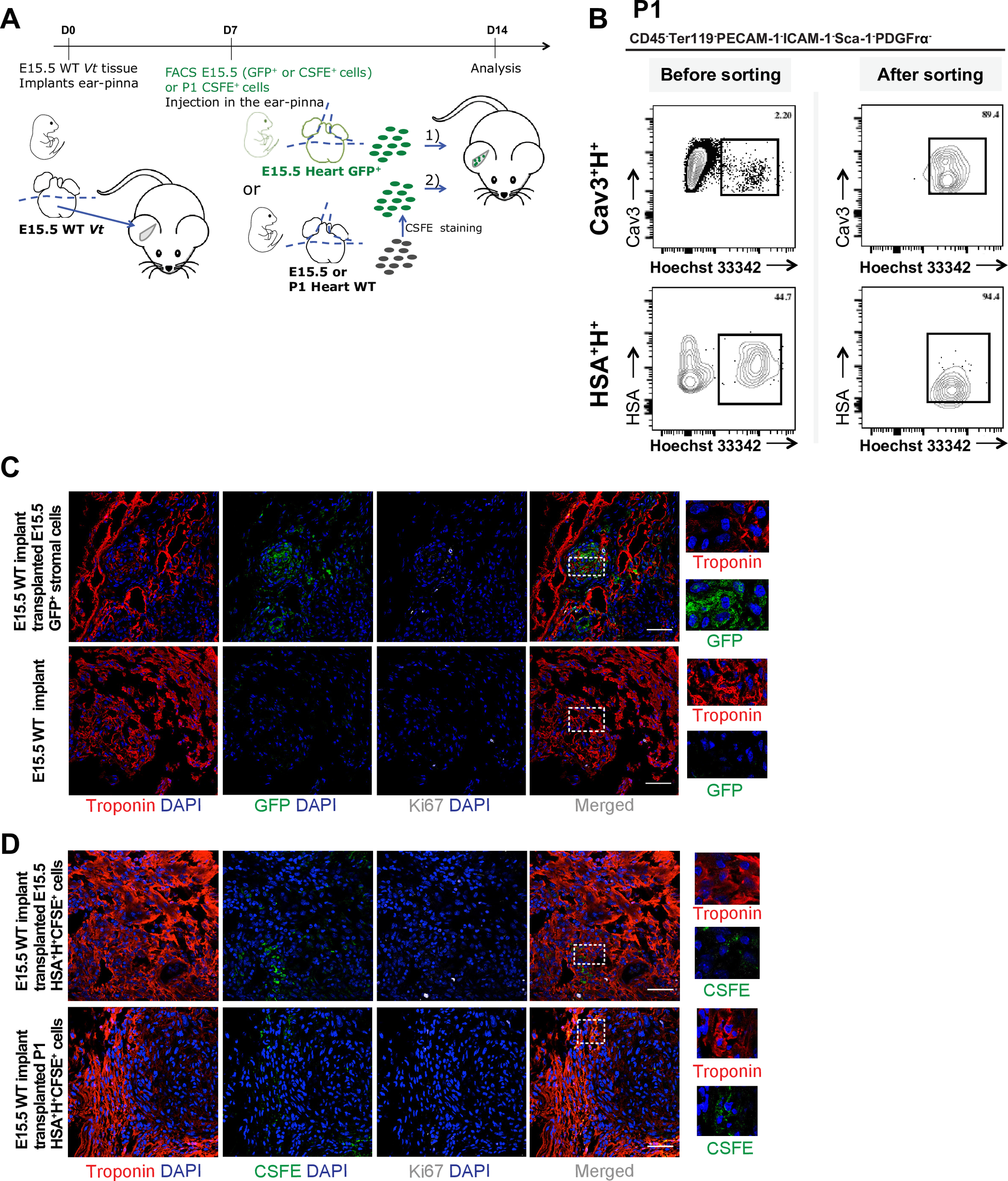

Supplement: S8 Fig — (A) Schematic representation of the experimental design followed in the ear-pinna experiments. (B) Sorting strategy for the isolation of HSA+H+ and Cav3+H+ for transplantation (right panels). Purity of the transplanted populations (left panels). (C) Immunohistochemistry analysis for the expression of troponin (red), Ki67 (white), DAPI (blue), and GFP (Ub–GFP cells) of embryonic cardiac tissue implants injected with cardiac stromal cells from the E 15.5 Ub–GFP mice (upper panels, first strategy) and implants not injected as controls for the experiment described in Fig 4G (lower panels). Higher magnification of the region delimitated by the white rectangle (right panels). (D) Immunohistochemistry analysis for the expression of troponin (red), Ki67 (white), DAPI (blue), and CFSE (green) of embryonic cardiac tissue implants injected with E 15.5 or P1 WT HSA+H+CFSE+ cells (second strategy). Higher magnification of the region delimitated by the white rectangle (right panels). Scale bar: 50 μm. Cav3, Caveolin-3; CFSE, carboxyfluorescein succinimidyl ester; E, embryonic day; GFP, green fluorescent protein; HSA, heat stable antigen; Ki67, Kiel clone 67; Ub–GFP, Ubiquitin–GFP; WT, wild type. (TIF) [file pbio.3000335.s008.tif]

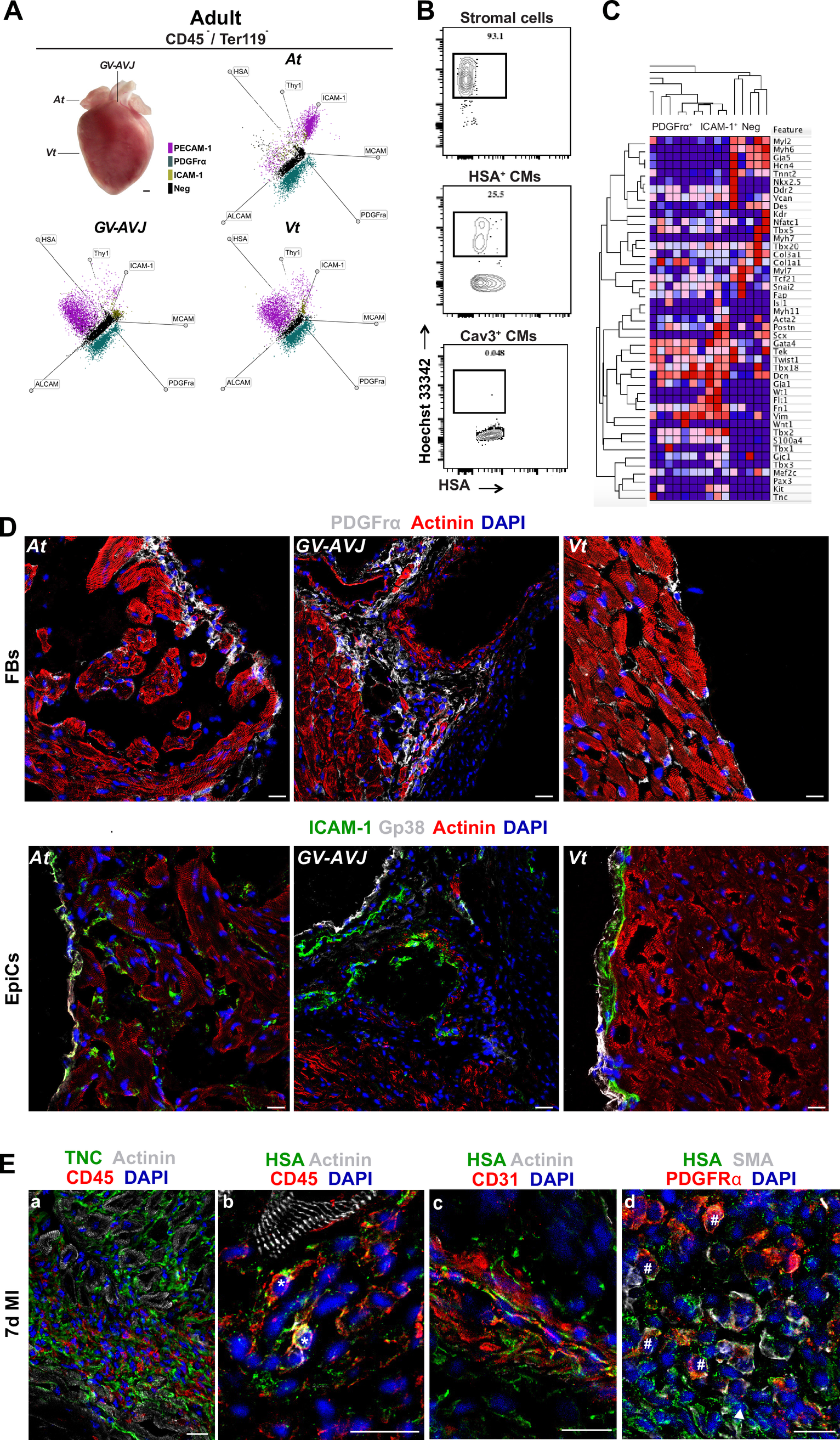

Supplement: S9 Fig — (A) Macroscopic view of adult heart, depicting the dissected cardiac regions: At, GV-AVJ, and Vt. Scale bar: 1 mm. Radar plots of flow cytometry analysis in the CD45− and Ter119− cell fraction for the surface expression of HSA, Thy1, PECAM-1, ICAM-1, MCAM, ALCAM, and PDGFrα in the indicated heart regions (n = 2). (B) Flow cytometry profiles of adult whole-heart suspensions (the great vessels were dissected out) stained with Cav3, HSA, and Hoechst 33342, after exclusion of CD45-, Ter119-, PECAM-1−, PDGFrα-, and ICAM-1−expressing cells. Gating strategy and Hoechst 33342 (H+) expression in stromal cells (ICAM-1+, upper panel), HSA+ CMs (middle panel), and Cav3+ CMs (lower panel). (C) Heat map displays the unsupervised hierarchical clustering analysis of the multiplex qRT-PCR data in 100 sorted cells of the indicated adult cardiac populations (n = 3). (D) Representative adult heart sections of the 3 heart regions (At, GV-AVJ, and Vt) stained for Actinin (red) and nuclear content (DAPI; blue), showing (1) FBs (PDGFrα+ cells; top row), (2) EpiCs (Gp38+ cells), and (3) EPDCs (ICAM-1+ cells); bottom rows. Scale bar: 20 μm. (E) Representative adult heart sections 7 days post MI stained for Actinin (red) and nuclear content (DAPI; blue), showing (a) the cellular infiltrate in the peri-infarcted region (CD45+ hematopoietic cells and the extracellular matrix protein TNC); and coexpression of HSA with (b) hematopoietic cells (CD45+), (c) ECs (PECAM-1+), and (d) SMCs (SMA+PDGFrα+); scale bar: 20 μm. The underlying data in (B) can be found within S8 Data. ALCAM, activated leukocyte cell adhesion molecule; At, atria; Cav3, Caveolin-3; CD45, cluster of differentiation 45; CM, cardiomyocyte; EC, endothelial cell; EPDC, epicardial-derived cell; EpiC, epicardial cell; FB, fibroblast; Gp38, glycoprotein 38; GV-AVJ, great vessels and atrioventricular junction; HSA, heat stable antigen; ICAM-1, intercellular adhesion molecule 1; MCAM, melanoma cell adhesion molecule; MI, myocardial infarc [file pbio.3000335.s009.tif]
