# Supplementary material for: Mouse HSA+ immature cardiomyocytes persist in the adult heart and expand after ischemic injury
Source: PLoS Biol. 2019 Jun 27;17(6):e3000335. doi: 10.1371/journal.pbio.3000335 (PMC6619826; doi:10.1371/journal.pbio.3000335)
Supplement: S1 Table — (DOCX) [file pbio.3000335.s010.docx]

| **Protein** | | | **Clone** | **Fluorochrome** | **Source** | **Reference** |
| --- | --- | --- | --- | --- | --- | --- |
| Caveolin 3 | Cav3 | | - | - | BD Biosciences | 610420 |
| CD146 | MCAM | | ME-9F1 | FITC | Miltenyi biotec | 130-102-230 |
| CD166 | ALCAM | | eBioALC48 | APC | eBiscience | 17-1661-82 |
| CD24 | HSA | | M1/69 | PECy7 | BD Bioscience | 560536 |
| CD31 | PECAM-1 | | MEC13.3 | Alexa Fluor 647 | BioLegend | 102516 |
| CD31 | PECAM-1 | | MEC13.3 | PE | BD Bioscience | 553373 |
| CD31 | PECAM-1 | | MEC13.3 | BV421 | BD Bioscience | 562939 |
| CD45 | - | | 30-F11 | PE | BioLegend | 103106 |
| CD45 | - | | 30-F11 | B | BioLegend | 103104 |
| CD54 | ICAM-1 | | YN1/1.7.4 | PB | BioLegend | 116116 |
| CD54 | ICAM-1 | | 3E2 | B | BD Bioscience | 553251 |
| CD90.2 | Thy1 | | 30-H12 | FITC | BD Bioscience | 553013 |
| CD90.2 | Thy1 | | 30-H12 | PE | BD Bioscience | 553014 |
| CD90.2 | Thy1 | | 53-2.1 | BV605 | BD Bioscience | 563008 |
| c-Kit | - | | 2B8 | APCCy7 | BioLegend | 105825 |
| Gp38 | - | | eBio8.1.1 (8.1.1) | eFluor 660 | eBiscience | 50-5381-82 |
| Ki67 | - | | MOPC-21 | FITC | BD Bioscience | 556026 |
| PDGFrα | - | | APA5 | PE | BioLegend | 135905 |
| Sca-1 | - | | D7 | PECy5 | BioLegend | 108110 |
| Ter119 | - | | TER-119 | PE | BioLegend | 116208 |
| Ter119 | - | | TER-119 | B | BD Bioscience | 553672 |
| TroponinT | Tnnt | | 13-11 | - | Thermo Scientific | MS-295-P0 |
|  | | Conjugated-secondary antibodies and – strepatavidin | | | | |
| Donkey Anti-Mouse IgG |  | | - | Cy3 | Jackson Immunoreserach Laboratories | 715-167-003 |
| SAV |  | | - | APCCy7 | BioLegend | 405208 |
| SAV |  | | - | BV421 | BioLegend | 405225 |
