# Supplementary material for: Mouse HSA+ immature cardiomyocytes persist in the adult heart and expand after ischemic injury
Source: PLoS Biol. 2019 Jun 27;17(6):e3000335. doi: 10.1371/journal.pbio.3000335 (PMC6619826; doi:10.1371/journal.pbio.3000335)
Supplement: S2 Table — (DOCX) [file pbio.3000335.s011.docx]

| **Primary antibodies** | | | | | |
| --- | --- | --- | --- | --- | --- |
| **Protein** | | **Isotype** | **Conditions** | **Source** | **Reference** |
| Actinin | - | Mouse IgG | 1:300 | Sigma | A7811 |
| α-Smooth Muscle Actin | SMA | Mouse IgG | 1:300 | Sigma | A5228 |
| Caveolin3 |  | Mouse IgG | 1:150 | BD Bioscience | 610420 |
| CD166 | ALCAM | Rat IgG | 1:50 | eBiscience | 17-1661-82 |
| CD24 | HSA | Rat IgG | 1:150 | eBiscience | 14-0242-81 |
| CD31 | PECAM-1 | Goat IgG | 1:250 | SCBT | sc-1506 |
| CD54 | ICAM-1 | Rat IgG | 1:300 | eBioscience | 14-0541 |
| Gp38 | - | Hm IgG | 1:300 | Novus | NB600-1015SS |
| Ki67 | - | Rabbit IgG |  | Abcam | ab15580 |
| Laminin | - | Rabbit IgG | 1:500 | Sigma | L9393 |
| PDGFrα | - | Goat IgG | 1:500 | R&D Systems | AF1062 |
| Sca-1 | - | Rat IgG |  | BD Pharmingen | 553333 |
| **Secondary antibodies** | | | | | |
| **Antibody** | | | **Fluorochrome** | **Source** | **Reference** |
| Donkey Anti-Mouse IgG | | | Cy3 | Jackson Immunoreserach Laboratories | 715-167-003 |
| Chick Anti-Mouse IgG | | | 647 | Invitrogen | A-21463 |
| Donkey Anti-Rat IgG | | | 488 | Invitrogen | A-21208 |
| Donkey Anti-Goat IgG | | | 594 | Invitrogen | A-11058 |
| Donkey Anti-Rabbit IgG | | | 568 | Invitrogen | A10042 |
| SAV | | | PECy7 | BioLegend | 405206 |
