# Supplementary material for: Mouse HSA+ immature cardiomyocytes persist in the adult heart and expand after ischemic injury
Source: PLoS Biol. 2019 Jun 27;17(6):e3000335. doi: 10.1371/journal.pbio.3000335 (PMC6619826; doi:10.1371/journal.pbio.3000335)
Supplement: S3 Table — (DOCX) [file pbio.3000335.s012.docx]

| **Gene** | **Gene Symbol** | **Gene ID** | **Taqman Assay ID** |
| --- | --- | --- | --- |
| *Actin beta* | *Actb* | 11461 | Mm00607939_s1 |
| *Arnt* | *Arnt* | 11863 | Mm00507836_m1 |
| *aMHC* | *Myh6* | 17888 | Mm00440359_m1 |
| *bMHC* | *Myh7* | 140781 | Mm00600544_m1 |
| *Caveolin 3* | *Cav3* | 12391 | Mm01182632_m1 |
| *Casq1* | *Casq1* | 12372 | Mm00486725_m1 |
| *Cdkn1a* | *Cdkn1a* | 12575 | Mm00432448_m1 |
| *c-Kit* | *Kit* | 16590 | Mm00445212_m1 |
| *Ckm* | *Ckm* | 12715 | Mm01321487_m1 |
| *Cited1* | *Cited1* | 12705 | Mm01235642_g1 |
| *Coll I* | *Col1a1* | 12842 | Mm00801666_g1 |
| *Coll III* | *Col3a1* | 12825 | Mm01254476_m1 |
| *Connexin 43* | *Gja1* | 14609 | Mm00439105_m1 |
| *Connexin 40* | *Gja5* | 14613 | Mm01265686_m1 |
| *Connexin 45* | *Gjc1* | 14615 | Mm01253027_m1 |
| *Cox8b* | *Cox8b* | 12869 | Mm00432648_m1 |
| *cTnt* | *Tnnt2* | 21956 | Mm01290256_m1 |
| *DDR2* | *Ddr2* | 18214 | Mm00445615_m1 |
| *Decorin* | *Dcn* | 13179 | Mm00514535_m1 |
| *Desmin* | *Des* | 13346 | Mm00802455_m1 |
| *Egln1* | *Egln1* | 112405 | Mm00459770_m1 |
| *Fap* | *Fap* | 14089 | Mm01329177_m1 |
| *Fhl1* | *Fhl1* | 14199 | Mm04204611_g1 |
| *Fibronectin* | *Fn1* | 14268 | Mm01256744_m1 |
| *Flk1* | *Kdr* | 16542 | Mm01222421_m1 |
| *Flt1* | *Flt1* | 14254 | Mm01210866_m1 |
| *Fsp1* | *S100a4* | 20198 | Mm00803371_m1 |
| *Gapdh* | *Gapdh* | 14433 | Mm99999915_g1 |
| *Gata4* | *Gata4* | 14463 | Mm00484689_m1 |
| *HCN4* | *Hcn4* | 330953 | Mm01176086_m1 |
| *Hif1a* | *Hif1a* | 15251 | Mm00468869_m1 |
| *Hopx* | *Hopx* | 74318 | Mm00558630_m1 |
| *HPRT* | *Hprt* | 15452 | Mm01545399_m1 |
| *Islet1* | *Isl1* | 16392 | Mm00517585_m1 |
| *Mef2c* | *Mef2c* | 17260 | Mm01340842_m1 |
| *Mgp* | *Mgp* | 17313 | Mm00485009_m1 |
| *Mki67* | *Mki67* | 17345 | Mm01278617_m1 |
| *Mlc2v* | *Myl2* | 17906 | Mm00440384_m1 |
| *Mlc2a* | *Myl7* | 17898 | Mm01183005_g1 |
| *Mt1* | *Mt1* | 17748 | Mm00496660_g1 |
| *Myl1* | *Myl1* | 17901 | Mm00659043_m1 |
| *Myl9* | *Myl9* | 98932 | Mm01251442_m1 |
| *Nfatc1* | *Nfatc1* | 18018 | Mm00479445_m1 |
| *NG2* | *Vcan* | 13003 | Mm01283063_m1 |
| *Nkx2.5* | *Nkx2-5* | 18091 | Mm01309813_s1 |
| *Scleraxis* | *Scx* | 20289 | Mm01205675_m1 |
| *Slc2a1* | *Slc2a1* | 20525 | Mm00441480_m1 |
| *Slc2a4* | *Slc2a4* | 20528 | Mm00436615_m1 |
| *Sln* | *Sln* | 66402 | Mm00481536_m1 |
| *Slug* | *Snai2* | 20583 | Mm00441531_m1 |
| *SMA* | *Acta2* | 11475 | Mm01546133_m1 |
| *sm-MHC* | *Myh11* | 17880 | Mm00443013_m1 |
| *Otud7b* | *Otud7b* | 229603 | Mm01256852_m1 |
| *Pax3* | *Pax3* | 18505 | Mm00435491_m1 |
| *Periostin* | *Postn* | 50706 | Mm00450111_m1 |
| *Tbx1* | *Tbx1* | 21380 | Mm00448949_m1 |
| *Tbx2* | *Tbx2* | 21385 | Mm00436915_m1 |
| *Tbx3* | *Tbx3* | 21386 | Mm01195726_m1 |
| *Tbx5* | *Tbx5* | 21388 | Mm00803518_m1 |
| *Tbx18* | *Tbx18* | 76365 | Mm00470177_m1 |
| *Tbx20* | *Tbx20* | 57246 | Mm00451515_m1 |
| *Tcf21* | *Tcf21* | 21412 | Mm00448961_m1 |
| *Tenascin C* | *Tnc* | 21923 | Mm00495662_m1 |
| *Tie2* | *Tek* | 21687 | Mm00443243_m1 |
| *Tnnt1* | *Tnnt1* | 21955 | Mm00449089_m1 |
| *Twist1* | *Twist1* | 22160 | Mm04208233_g1 |
| *Ubb* | *Ubb* | 22187 | Mm01622233_g1 |
| *Vhl* | *Vhl* | 22346 | Mm00494137_m1 |
| *Vimentin* | *Vim* | 22352 | Mm01333430_m1 |
| *Wnt1* | *Wnt1* | 22408 | Mm01300555_g1 |
| *Wt-1* | *Wt1* | 22431 | Mm01337048_m1 |
